# Continuous Evolution System Based on Toxin–Antitoxin System Combined with Base Deaminase Accelerates the In Vivo Modification of Target Proteins

**DOI:** 10.1002/advs.76924

**Published:** 2026-08-20

**Authors:** Xinyu Zhang, Zhanzhi Liu, Ying Xu, Mingjing Sun, Guangzhen Zhou, Deming Rao, Xiaoqian Chen, Sheng Wang, Zhigang Li, Yan Huang, Jing Wu

**Affiliations:** ^1^ School of Biotechnology and Key Laboratory of Industrial Biotechnology, Ministry of Education Jiangnan University Wuxi Jiangsu Province China; ^2^ State Key Laboratory of Food Science and Resources Wuxi Jiangsu Province China; ^3^ International Joint Laboratory on Food Safety Jiangnan University Wuxi Jiangsu Province China

**Keywords:** base deaminase, in vivo continuous evolution, T7 RNA polymerase, toxin–antitoxin, transcription factor PsiR

## Abstract

To overcome the labor‐intensive nature of traditional directed evolution, the promoted *Escherichia coli*‐assisted continuous evolution (PEACE) system has been developed. PEACE 1.0 employs a cytidine deaminase‑T7 RNA polymerase (T7 RNAP) fusion for targeted mutagenesis and integrates a toxin–antitoxin selection module for spontaneous, growth‑coupled enrichment of evolved variants. Through dual optimization of mutagenesis and selection, PEACE 2.0 achieves an average mutation frequency of 3.49 × 10^−3^ per base pair within a 60‑h cycle (compared to 1.54 × 10^−3^ in 120 h for PEACE 1.0), while shortening the cycle reduces host background mutation accumulation. A dual‑factor (growth‑fluorescence) selection/screening mechanism eliminates a 12%–20% absolute fraction of undesired non‑specific survivors, and coupling with fluorescence‑activated cell sorting enables ultrahigh‑throughput screening exceeding 10^6^ variants per day. PEACE rapidly evolves T7 RNAP to recognize non‑canonical promoters and efficiently reprograms the transcription factor PsiR from a *d*‑psicose inducer to a *d*‑fructose/*d*‑mannose repressor, establishing a novel regulatory mode. These results demonstrate that PEACE is an efficient, versatile, and robust platform for in vivo continuous evolution with broad potential in protein engineering.

## Graphical abstract

The promoted *Escherichia coli*‐assisted continuous evolution (PEACE) system operates through a stringent three step genetic circuit: target gene mutation via a deaminase–T7 RNA polymerase (T7 RNAP) fusion, coupling variant function to antitoxin expression, and growth based selection of surviving cells. The optimized PEACE 2.0 runs on a 60‐h cycle, adds fluorescence based screening, and reprograms T7 RNAP and the transcription factor PsiR.

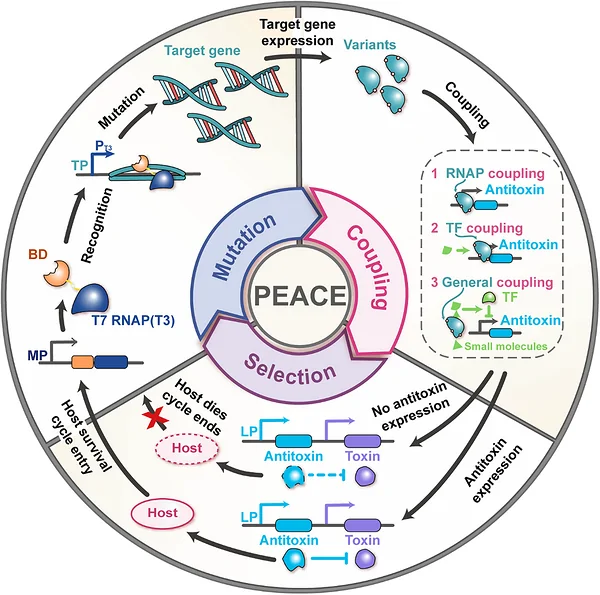

## 1 Introduction

Enzymes are indispensable biocatalysts that mediate most reactions in biological systems [1]. However, the native properties of natural enzymes—such as catalytic activity, specificity, stability, and expression levels—are often difficult to tailor directly to stringent industrial demands, which limits their utilization in commercial applications [2, 3]. To improve enzyme performance, various protein engineering techniques have been developed [4]. Among these, directed evolution can efficiently tune enzyme properties under defined selective pressures to meet diverse application needs [5, 6]. In traditional directed evolution, (ultra)high‐throughput screening/selection is required to identify the most promising variants from a target protein library constructed via genetic diversification methods such as random mutagenesis [7] and DNA shuffling [8]. As a multi‐stage manual process, each round of directed evolution involves in vitro library construction, transformation into an appropriate host cell, high‐throughput screening/selection, and variant identification, making it costly in both labor and time [1]. In contrast, in vivo continuous evolution enables the construction of a protein library and the screening/selection of variants to be carried out within the same host cell, thereby significantly reducing both the time and labor required [9, 10].

Among existing in vivo continuous evolution systems, *Escherichia coli* has been extensively studied as an ideal host owing to its well‑characterized genetic background, short life cycle, and mature genetic manipulation tools [11, 12]. Previous studies have provided various methods for introducing random mutations into *E. coli*, including the incorporation of mutated genes such as *mutD5* [13] and the use of the mutation‐prone strain *E. coli* XL1‐Red as a host [14]. In 2011, Esvelt et al. [15] proposed the phage‐assisted continuous evolution (PACE) platform, which combines multiple mutagenic elements to constitute the mutagenesis plasmid MP6 and couples mutation to phage infectivity through *pIII* expression [15, 16, 17]. Although the aforementioned mutagenesis methods have been successfully applied in various contexts, they predominantly mutagenize the entire genome, which indirectly reduces the effective mutation frequency on the genes of interest (GOIs) and may produce unexpected mutations that compromise genotype–phenotype coupling. Therefore, in most cases, GOI‐focused mutagenesis is desirable to advance the practical application of in vivo continuous evolution.

To focus mutagenesis on GOIs in vivo, various toolkits have been developed, including error‐prone DNA polymerase I (Pol I) variants [18, 19], orthogonal error‐prone DNA polymerases applied to bacterial continuous evolution systems [20, 21], base editors (BEs) that employ dead Cas9 (dCas9) and base deaminase (BD) [22, 23, 24], and BD‐T7 RNA polymerase (T7 RNAP) fusion proteins capable of precisely targeting and inducing mutations in the GOIs [25, 26, 27]. Among these strategies, the MutaT7 system developed by Moore et al. [27], which is based on the fusion of a BD and T7 RNAP, has become an important platform for in vivo target mutagenesis by confining mutations within a defined transcriptional unit. The initial system utilized a cytidine deaminase (CD)‐T7 RNAP fusion, and a common auxiliary strategy to enhance its efficiency involves inhibiting the base excision repair (BER) pathway, either by knocking out the host uracil DNA glycosylase (UNG) or by introducing the UNG inhibitor (UGI), thereby significantly increasing the C:G > T:A mutation rate. In terms of editing precision and controllability, the original MutaT7 design employed multiple T7 terminators to focus mutations within the open reading frame of the GOIs [27]. Subsequently, the T7‐DIVA system developed by Álvarez et al. [26] further improved controllability by coupling dCas9, allowing flexible adjustment of the editing window length. In recent studies, expanding the mutational spectrum has become a key direction for optimization. For example, Mengiste et al. [28] and Seo et al. [29] introduced adenosine deaminase (AD) to create Dual7/8 and eMutaT7^transition^, respectively, extending the inducible mutation types from C:G > T:A to A:T > G:C, which enhances the platform's capacity for diversified mutagenesis. In addition, Mengiste et al. [30] reported a next‑generation MutaT7^GDE^ system based on a single chimeric enzyme capable of efficiently and uniformly installing all possible transition mutations, further expanding the utility of MutaT7 tools for in vivo continuous evolution. Furthermore, Wang et al. [31] developed a MutaT7‐based platform in *Bacillus subtilis*, and Zhu et al. [32] established a MutaT7 system in *Vibrio natriegens*, thereby expanding the application scope of MutaT7 from a host perspective. In terms of GOI selection, Deng et al. [33] made a breakthrough by using MutaT7 to successfully evolve the growth‐coupled protein CelB, which provides support for integrating MutaT7 with growth‐dependent selection in the future. These teams have laid a solid foundation for applying MutaT7 in GOI evolution. Despite this progress, current MutaT7 studies have primarily focused on GOIs that are either directly observable, such as eGFP, or directly linked to host growth, such as antibiotic resistance or growth‐coupled proteins, thus limiting the application of MutaT7. To broaden the application scope of MutaT7 in the field of in vivo continuous evolution, pairing it with suitable screening/selection methods represents a viable direction for further development.

To address this general limitation in versatility, we developed a versatile new in vivo continuous evolution system in *E. coli*, termed the promoted *E. coli*‐assisted continuous evolution (PEACE) system. PEACE distinguishes itself by coupling a MutaT7‐based mutagenesis element with a toxin–antitoxin system (TAS)‐based selection element, thereby establishing a stringent logical linkage between the desired protein function and cell survival. As the initial version, PEACE 1.0 uses a human activation‐induced cytidine deaminase (AID)‐T7 RNAP fusion protein as the mutagenic element. Notably, unlike the later optimized versions of the MutaT7 system, PEACE 1.0 retains the host's native BER pathway (without UNG knockout or UGI addition) to explore its potential effect on mutational diversity, albeit at a lower mutation frequency. The selection element is a TAS that intrinsically couples antitoxin expression to the properties of the desired variants, enabling continuous, autonomous enrichment without requiring external additives such as antibiotics or specific nutrients. Subsequently, T7 RNAP was selected as the GOI to validate the feasibility of PEACE, and this approach successfully generated P_CTGA_ [34]‐responsive variants, demonstrating the capacity of PEACE 1.0 for GOI evolution. However, PEACE 1.0 was hampered by a low mutation rate and a high false‐positive rate. To overcome these limitations, we engineered both the mutagenesis and screening/selection elements, leading to an optimized system termed PEACE 2.0. In the mutagenesis element, PEACE 2.0 employs an optimized BD combination: CD PmCDA1 with UGI to enhance the C:G > T:A mutation frequency, and concurrently incorporates the adenosine deaminase TadA^9e^—selected for its high editing efficiency and precise targeting window [35]—to introduce A:T > G:C mutations, thereby achieving both a high mutation rate and a broadened mutational spectrum. Concurrently, to improve screening stringency and efficiency, the original TAS was augmented with eGFP, creating a dual‐factor (growth‐fluorescence) screening mechanism. This not only significantly reduces false positives but also enables ultra‐high‐throughput sorting via fluorescence‑activated cell sorting (FACS) after evolution, further minimizing manual intervention. The evolutionary results using T7 RNAP as the GOI demonstrate that PEACE 2.0 can generate more desired variants than PEACE 1.0 in a shorter time frame. Subsequently, we applied PEACE 2.0 to the continuous evolution of the transcription factor PsiR, which can respond to *d*‐allulose, and successfully engineered the allosteric regulatory mechanism of PsiR, thereby reprogramming the biosensing mode of this factor from *d*‐allulose‐dependent activation to *d*‐fructose/*d*‐mannose‐dependent inhibition. PEACE 2.0 introduces a simple‐operation and precisely controllable protein continuous evolution paradigm, resolving the problems of operational complexity and limited controllability. It provides a robust platform for reprogramming transcription‑related proteins, representing a key step toward engineering a broader range of proteins (e.g., metabolic enzymes) by coupling their activities to the selection system via designed biosensors.

## 2 Results

### 2.1 Design and Principle of PEACE 1.0

**FIGURE 1:**
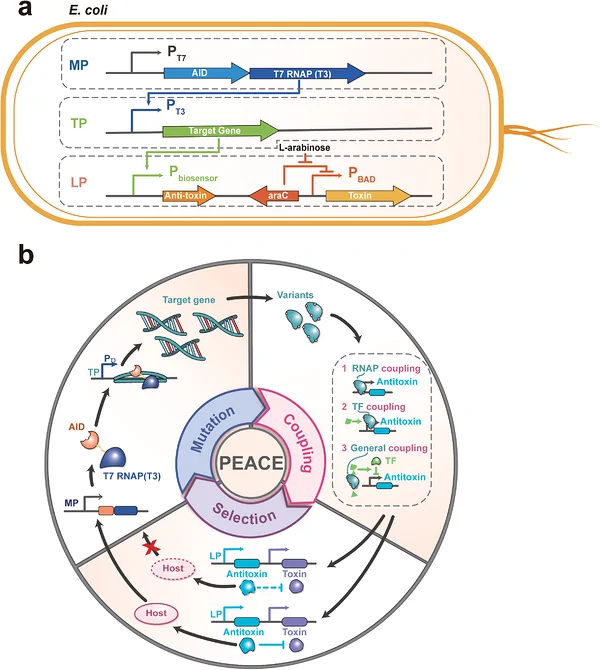
Schematic diagram of the PEACE system. (a) The PEACE system consists of three plasmids: the mutagenesis plasmid (MP), the target plasmid (TP), and the lethal plasmid (LP). (b) Continuous evolution is divided into three main stages: mutation, coupling, and screening/selection. Mutation: the MP introduces genetic diversity into the target gene. Coupling: the desired variant initiates antitoxin expression either directly or indirectly through a biosensor that responds to the conversion product. Screening/selection: under controlled expression of the toxin as the selection pressure, sufficient expression of the antitoxin neutralizes the lethal effect of the toxin on the host cell, allowing cell survival; conversely, absent or insufficient expression of the antitoxin leads to host cell death.

To expand the toolkit for in vivo continuous evolution and promote the application of directed evolution, PEACE 1.0 was designed and constructed. Using *E. coli* as the host, the system consists of a mutagenesis plasmid (MP), a target plasmid (TP), and a lethal plasmid (LP), as illustrated in Figure 1a. The in vivo mutagenesis of the GOI is achieved by the AID‐T7 RNAP variant (T3) fusion protein encoded by MP‐AID (Table S2). The T7 RNAP (T3) specifically recognizes the T3 promoter (P_T3_) on the target plasmid, enabling targeted transcription of the downstream GOI [34]. To ensure the convenience and versatility of the system, PEACE employs the engineered *E. coli* strain BL21(DE3)—widely used for heterologous protein expression—whose genome already contains an integrated T7 RNAP gene. Therefore, the fusion protein on MP‐AID is directly expressed under the control of the T7 promoter (P_T7_) on the plasmid. Given the strong activity of the P_T7_, which may cause cytotoxic overexpression of the fusion protein, the growth profile of the host was monitored in subsequent evolution experiments to ensure that the mutagenic element did not lead to growth impairment. During transcription, the fused AID induces in vivo mutagenesis of the GOI by converting cytosine (C) to uracil (U) on the exposed single‐stranded DNA. This conversion is predominantly fixed as thymine (T) through DNA replication, whereas BER‐mediated repair can occasionally introduce other base substitutions at a lower frequency [36].

In addition, the selection coupled to host cell growth is achieved by the TAS in the LP. In the TAS, the gene encoding the toxin is transcribed from the P_BAD_, which is induced by *l*‐arabinose, while that of the antitoxin is controlled by a specific biosensor that responds to the properties of the target variants. The desired variant can directly or indirectly initiate antitoxin expression, thereby antagonizing the toxin. Under continuous toxin expression as the selection pressure, host cells harboring the desired variant can survive in continuous culture, whereas non‐evolved cells are eliminated (Figure 1b).

### 2.2 Construction and Characterization of MP‐AID

**FIGURE 2:**
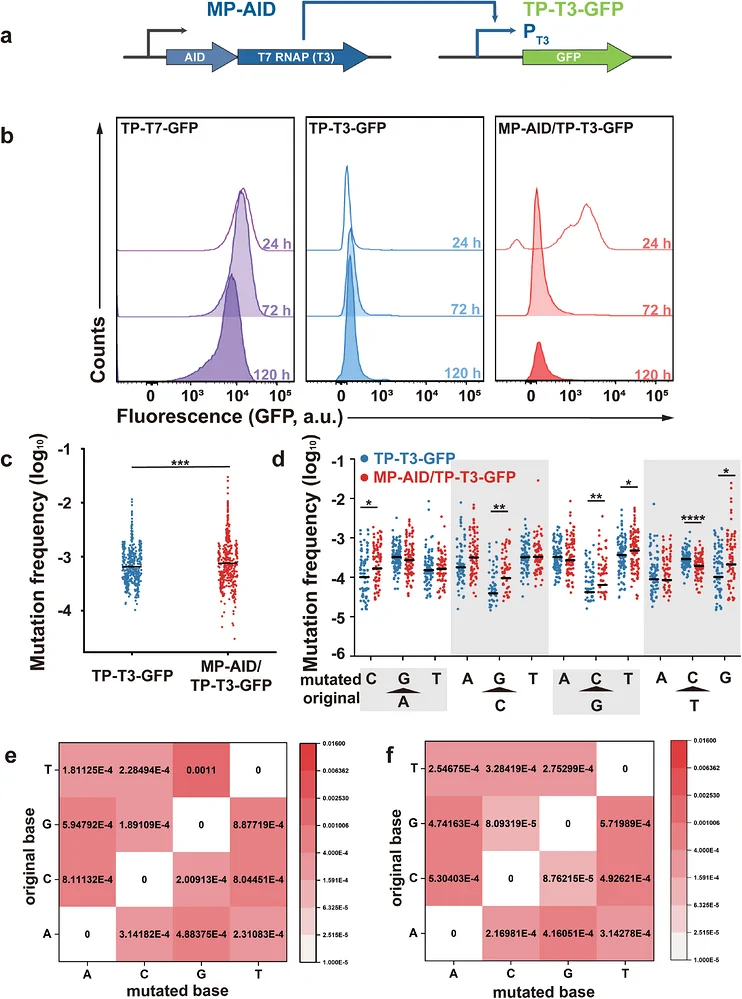
Characterization of the mutagenic activity of MP‐AID. (a) Schematic of the experimental design used to characterize the AID‐T7 RNAP (T3) fusion protein with eGFP as the reporter. (b) Fluorescence signals recorded by flow cytometry after continuous evolution. *E. coli* BL21(DE3)/MP‐AID/TP‐T3‐GFP served as the experimental group, while *E. coli* BL21(DE3)/TP‐T7‐GFP and *E. coli* BL21(DE3)/TP‐T3‐GFP were used as the positive and negative controls, respectively. (c) Mutation frequencies of *E. coli* BL21(DE3)/TP‐T3‐GFP and *E. coli* BL21(DE3)/MP‐AID/TP‐T3‐GFP. Mutation frequency is defined as the ratio of mutated reads to the total sequencing depth at a given site. Statistical analysis was performed using one‑way ANOVA; *p* < 0.001 was considered significant. (d) Comparison of mutation frequencies for all possible single‐base substitution types between samples with and without MP‐AID. (e) Mutational spectrum of MP‐AID. (f) Mutational spectrum of TP‐T3‐GFP.

To prevent interference from the endogenous wild‐type T7 RNAP in the *E. coli* BL21(DE3) host and ensure orthogonality, we adopted an engineered switch. This switch consists of a fusion of a variant T7 RNAP (T3) to AID, which specifically recognizes the P_T3_ [34]. To validate the mutagenic potential of the AID‐T7 RNAP (T3) fusion protein and its orthogonality toward the P_T3_ within the MP‐AID, we selected eGFP as the GOI (Figure 2a). Three recombinant strains—the experimental group (MP‐AID with eGFP under the control of the P_T3_, MP‐AID/TP‐T3‐GFP (Table S2)), a positive control (eGFP under the constitutive P_T7_ promoter, TP‐T7‐GFP (Table S2)), and a negative control (eGFP under the P_T3_ promoter in the absence of MP‐AID, TP‐T3‐GFP)—were continuously cultured. FACS was used to record and analyze the fluorescence signals from each group of recombinant cells. The results demonstrate negligible crosstalk between from the host's native T7 RNAP and the P_T3_, as evidenced by a >100‐fold difference in fluorescence intensity between the TP‐T7‐GFP and TP‐T3‐GFP constructs (Figure 2b). The persistent low signal (0–10^2^ a.u.) from the P_T3_ over 120 h confirms that the genomically encoded T7 RNAP does not recognize or activate the P_T3_, thus ensuring that the transcriptional output of the GOI is exclusively and precisely controlled by MP‐AID. Figure 2b also confirms the successful deployment of MP‐AID: the robust eGFP expression (10^3^–10^4^ a.u.) at 24 h in the MP‐AID/TP‐T3‐GFP strain verifies effective binding and transcription initiation by the T7 RNAP (T3) at the P_T3_. In addition, a notable and unique phenomenon emerged: only in this group did the fluorescence intensity decline significantly as the continuous culture progressed beyond 24 h. This time‐dependent loss of signal, which was absent in all control groups, suggests that MP‐AID was performing its designed function‐introducing mutations into the GOI that ultimately ablated its fluorescence. It should be noted that although continuous expression of a base deaminase may impose certain stress on host growth, prolonged induction of MP‐AID did not lead to host growth inhibition in this study. The corresponding validation experiments are presented in Section 2.4 and Figure S3.

To quantify the mutagenic effect induced by MP‐AID, we performed next‐generation sequencing (NGS) to analyze the mutation frequency and spectrum of eGFP after 120 h of continuous evolution. The mutation frequency per base pair was calculated from the amplified GOI fragment (426 bp in length) of the MP‐AID/TP‐T3‐GFP strain, with the TP‐T3‐GFP strain serving as the control. Based on the NGS data, the mutation frequency at 120 h was determined as the ratio of mutated reads to total reads for each base position. The average mutation frequency in the experimental group reached 1.54 × 10^−3^ per base pair, representing a frequency approximately 1.5 times that of the control (1.03 × 10^−3^) (Figure 2c). Consistent with this finding, analysis of single‐site mutation frequencies along the entire 426 bp GOI fragment (Figure S1) revealed that the MP‐AID group exhibited elevated mutation frequencies at certain positions compared with the control. Analysis of all 12 possible single‐base substitution types showed that MP‐AID not only elevated the overall mutation burden but also promoted a diverse mutational spectrum. Specifically, the mutation frequency distributions for five substitution types (A > C, C > G, G > C, G > T, and T > G) were significantly higher in the experimental group than in the control (Figure 2d). Moreover, within the experimental group, the mutation frequencies across different substitution types were relatively uniform, ranging from 1.81 × 10^−4^ to 1.10 × 10^−3^ per base pair, indicating no strong mutational bias (Figure 2e). In contrast, the frequencies in the control group were predominantly lower, ranging from 8.09 × 10^−5^ to 5.72 × 10^−4^ (Figure 2f). These results confirm that MP‐AID, as developed in this study, facilitates targeted mutagenesis of the GOI with a broad spectrum that is not limited to C:G > T:A. Although the overall mutation frequency is relatively low, this aligns with the design rationale of PEACE 1.0—trading a higher mutation rate for a broader mutational spectrum by intentionally retaining the host's native BER pathway (without UNG knockout or UGI addition).

### 2.3 Construction and Characterization of LP

To establish an efficient growth‐coupled selection mode for in vivo continuous evolution, we adopted a TAS as the core of LP. This strategy directly links beneficial mutations in the GOI to the host's survival advantage, thereby enriching positively evolved clones. To this end, we selected four candidate TAS—CcdB/CcdA [37], PezT/PezA [38], SezT/SezA [39], and Zeta toxin/Epsilon [40]—for subsequent evaluation of their suitability in *E. coli*. We next designed a rigorous induction assay to functionally characterize these TAS. The assay comprised three complementary conditions: (i) selective induction of the toxin gene to evaluate its lethal potency, (ii) individual induction of the antitoxin gene to verify its biological safety, and (iii) co‐induction of both toxin and antitoxin genes to demonstrate effective neutralization and cellular protection. By comparing bacterial growth curves (0–5.5 h) under these conditions, we aimed to identify the most effective TAS for constructing a high‐performance LP.

**FIGURE 3:**
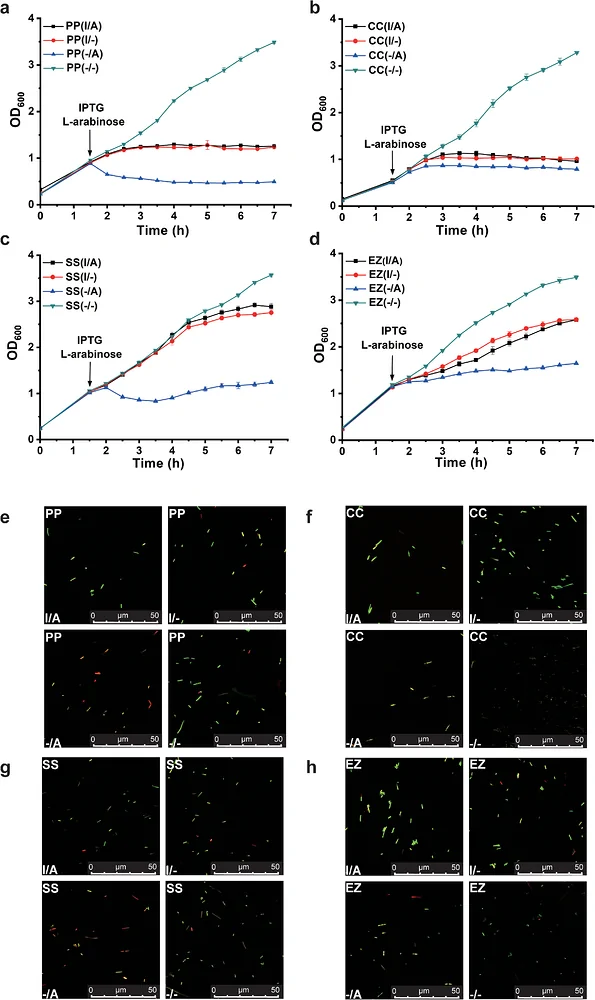
Influence of toxin–antitoxin systems on the host. (a–d) Growth curves of recombinant cells harboring PezT/PezA (a), CcdB/CcdA (b), SezT/SezA (c), and Zeta toxin/Epsilon (d). "
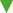
" means no inducer (‐/‐); "
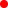
" means IPTG only, inducing antitoxin expression (I/‐); "
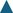
" means *l* ‐arabinose only, inducing toxin expression (‐/A); "
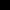
" means both IPTG and *l* ‐arabinose, co‐inducing antitoxin and toxin expression (I/A). (e–h) Fluorescence microscopy images of SYTO9/PI‑stained recombinant cells harboring PezT/PezA (e), CcdB/CcdA (f), SezT/SezA (g), and Zeta toxin/Epsilon (h). Scale bar, 50 µm. All images were acquired under identical instrument settings using the same objective lenses on the same day. Some images were proportionally enlarged to ensure uniform scale bar representation.

As shown in Figure 3a–d, among the four TAS tested, both PezT (Figure 3a) and CcdB (Figure 3b) exhibited strong growth inhibition when the toxin was induced alone (‐/A), whereas SezT (Figure 3c) and Zeta toxin (Figure 3d) showed relatively weaker effects. In the toxin–antitoxin co‐expression assay (I/A), only PezA significantly neutralized the toxicity of PezT (Figure 3a), leading to partial restoration of host growth; in contrast, CcdA provided almost no effective antagonism against CcdB (Figure 3b), with host growth remaining severely suppressed. Quantitative analysis further supported these observations. The final OD_600_ values for the four TAS pairs under different induction conditions were used to define three parameters: toxin inhibition rate = 1 − (OD_600_ of toxin‑only group/OD_600_ of uninduced group); co‑expression inhibition rate = 1 − (OD_600_ of the toxin+antitoxin group/OD_600_ of uninduced group); and recovery rate = toxin inhibition rate − co‑expression inhibition rate. Among the four pairs, PezT/PezA exhibited the highest toxin inhibition rate (85.83%) together with a substantial recovery rate of 21.93%. CcdB/CcdA showed a high toxin inhibition rate (75.99%) but a minimal recovery rate (5.33%), indicating poor antitoxin activity of CcdA against CcdB. SezT/SezA yielded the highest recovery rate (45.93%) but a relatively low toxin inhibition rate (65.21%), whereas the Zeta toxin/Epsilon pair displayed the lowest toxin inhibition rate (52.82%) and a recovery rate of 26.75%. Collectively, these quantitative comparisons support the selection of PezT/PezA as the optimal module for LP construction, owing to its strong lethality and effective antitoxin neutralization.

To more directly validate the lethality and neutralization efficacy of the TAS at the single‐cell level, and to exclude the possibility that host cells were merely growth‐arrested rather than lethally compromised, we performed SYTO9/propidium iodide (PI) staining on cells under four different induction conditions. SYTO9 and PI are fluorescent dyes commonly used to assess bacterial viability and membrane integrity, based on their differential permeability to intact versus damaged membranes. SYTO9, a green‐fluorescent nucleic acid stain, penetrates intact membranes and labels live cells, whereas PI, a red‐fluorescent stain, enters only cells with compromised membranes and labels dead cells. The stained samples were observed and imaged by confocal laser scanning microscopy (Figure 3e–h). The results for the PezT/PezA (PP) pair (Figure 3e) were highly consistent with the growth curve data: cultures induced for toxin alone (‐/A) showed predominantly red fluorescence (dead cells), while the other three conditions (I/A, I/‐, ‐/‐) displayed primarily green fluorescence (live cells). For the CcdB/CcdA pair (Figure 3f), the “‐/A” condition presented a mixture of green, yellow, and red cells, which was inconsistent with the strong growth inhibition by CcdB observed in the growth assays, suggesting that CcdB may primarily inhibit growth rather than cause cell death. In contrast, for the SezT/SezA (Figure 3g) and Zeta toxin/Epsilon (Figure 3h) pairs, a substantial portion, or even the majority, of cells under the “‐/A” condition remained green, correlating with the observable growth increase seen in their respective growth curves. Based on this dual validation from growth curves and fluorescence microscopy, we ultimately selected the best‐performing PezT/PezA pair as the key component for LP construction, and the resulting plasmid was designated LP‐PP (Table S2).

### 2.4 In Vivo Continuous Evolution of T7 RNAP

After separately verifying the performance of MP‐AID and LP‐PP, we proceeded to validate the feasibility of PEACE 1.0 using a protein with a well‑defined mechanism and established research value. T7 RNAP functions as a single‐subunit enzyme that directly catalyzes RNA synthesis and exhibits high specificity for P_T7_. Developing T7 RNAP variants with orthogonal specificities can therefore play an important role in synthetic biology [34]. Accordingly, T7 RNAP was chosen as the GOI for continuous evolution to obtain transcriptional activity toward the non‑canonical T7 promoter P_CTGA_. Previous work by Meyer et al. [34] showed that the transcriptional activity of wild‐type T7 RNAP on genes downstream of P_CTGA_ is less than 1% of that on genes downstream of P_T7_.

**FIGURE 4:**
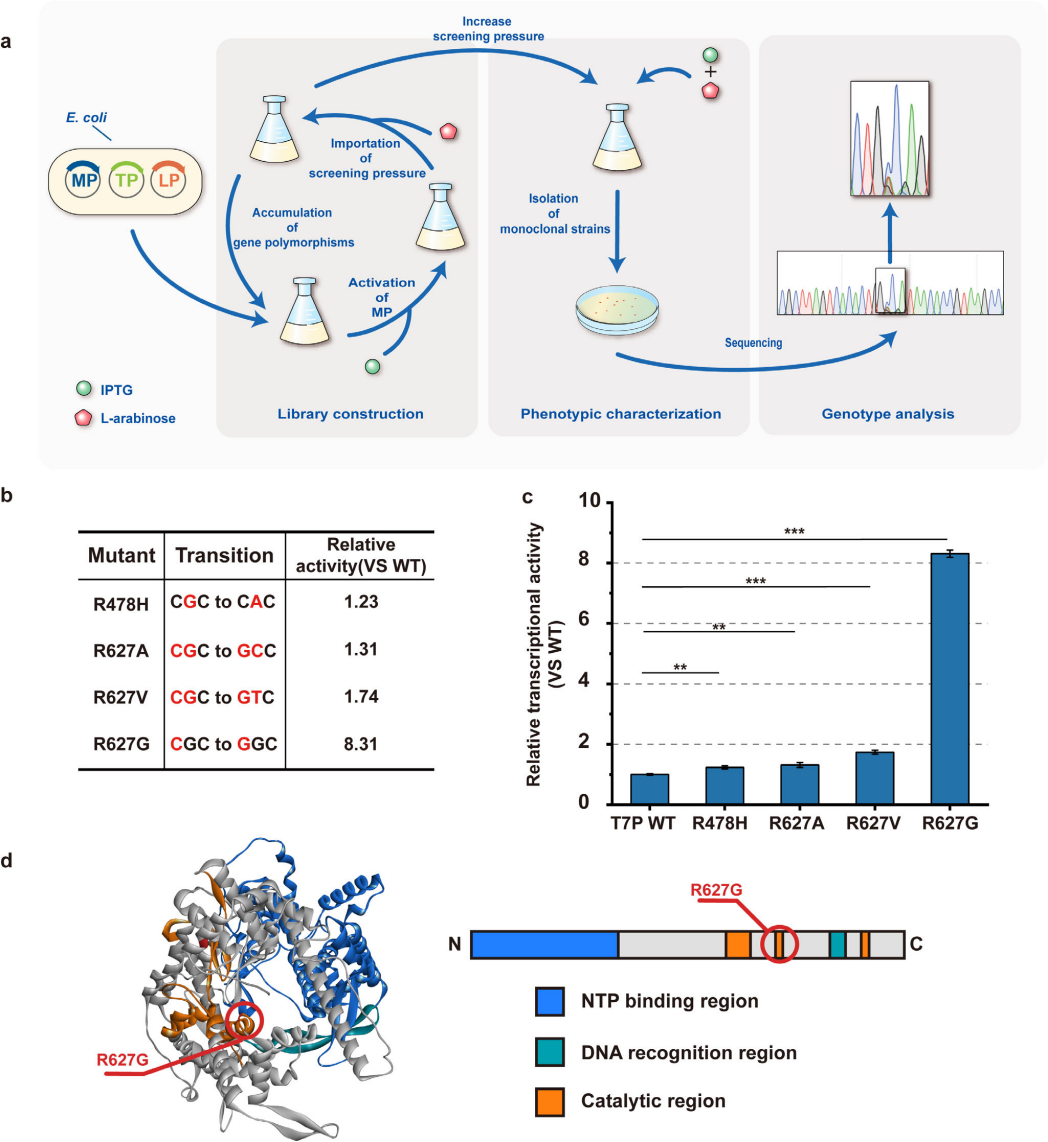
Validation of PEACE 1.0: continuous evolution of T7 RNAP. (a) Schematic diagram of the PEACE system for T7 RNAP evolution. Recombinant *E. coli* BL21(DE3) co‐transformed with MP‐AID, TP‐T7P, and LP‐RPP is continuously cultured in liquid medium containing the appropriate antibiotics. During cyclic culture, the inducers IPTG and *l*‐arabinose are added as indicated. After continuous culture, the surviving colonies are collected and subjected to genotypic analysis. (b) Summary of the T7 RNAP variants identified for P_CTGA_. Relative activity is defined as the activity of a variant toward P_CTGA_ relative to that of WT T7 RNAP toward the P_CTGA_. Values represent the mean of experiments performed in triplicate. (c) Transcriptional activity of WT T7 RNAP and the selected variants toward P_CTGA_. Activity was determined using *β*‐galactosidase as a reporter. Error bars represent the standard deviation of at least three independent replicates. Statistical significance was assessed by one‑way ANOVA; * indicates *p <* 0.05; ** indicates *p < *0.01; *** indicates *p <* 0.001. (d) Structural representation of the R627G mutation. The 3D structure (left) and 2D schematic (right) are color‐coded by functional domain: NTP binding domain (blue), DNA binding domain (teal), and catalytic domain (orange); the mutation site at position 627 is highlighted in red.

To direct the evolution toward the desired T7 RNAP phenotype, we constructed the recombinant plasmid, LP‐RPP (Table S2), in which the antitoxin gene is driven by the P_CTGA_ promoter. First, to determine whether wild‐type (WT) T7 RNAP could activate P_CTGA_, we monitored the growth kinetics of *E. coli* BL21(DE3)/LP‐RPP under various induction conditions over a 5.5‐h period. The results indicated that even upon addition of IPTG to induce WT T7 RNAP expression, the recombinant cells failed to grow normally (Figure S2), demonstrating that WT T7 RNAP lacks transcriptional activity toward P_CTGA_. Subsequently, the mutator element MP‐AID, the T7 RNAP expression element TP‐T7P (Table S2), and the selection element LP‐RPP were co‐transformed into *E. coli* BL21(DE3) to construct the PEACE 1.0 for T7 RNAP evolution. An overview of the evolution process is presented in Figure 4a. Prior to the formal evolution cycles, a pre‐cultivation step was conducted to confirm host fitness. The strain was subjected to five rounds of cultivation (120 h) with induction of only MP‐AID expression using IPTG, and the optical density was recorded at the beginning and end of each round. This confirmed that overexpression of the fusion protein from MP‐AID did not lead to significant growth inhibition (Figure S3). After continuous growth‐coupled selection, the final culture of the recombinant bacteria was plated on LB solid medium supplemented with the appropriate antibiotics and inducers, followed by overnight incubation at 37°C. Individual colonies were then picked and validated using KBseq (a whole‐plasmid sequencing technology based on high‐throughput sequencing; Sangon Biotech) and Sanger sequencing. The identified T7 RNAP variants are listed in Table S4. To verify the functionality of the selection system during evolution, we included two key controls in the initial PEACE 1.0 experiment: CK1 (with MP‑AID but without IPTG induction) and CK2 (lacking the mutagenesis plasmid). After evolution, only the experimental group yielded colonies on plates containing the toxin inducer, whereas both control groups did not, despite having cell densities comparable to or even higher than those of the experimental group (Figure S4). These results confirm that the selection system LP‑RPP functioned as designed.

To further characterize the functional properties of the selected T7 RNAP variants, we established an in vivo validation system employing *β*‐galactosidase as a reporter. The transcriptional efficiency of each T7 RNAP variant toward P_CTGA_ was indirectly assessed by measuring *β*‐galactosidase activity. As shown in Figure 4b,c, compared with the WT, the R478H, R627A, R627V, and R627G variants exhibited significantly enhanced transcriptional activity. Notably, the R627G variant showed an activity 8.31 times that of WT, demonstrating significantly enhanced transcriptional activity toward P_CTGA_.

Leveraging the resolved three‐dimensional structure of T7 RNAP, we performed a structure‐based analysis of the key residue R627G (Figure 4d). This site resides within the enzyme's catalytic domain (residues 626–642). In WT T7 RNAP, Arg627, with its positively charged extended side chain, establishes a rigid interaction network with adjacent residues, stabilizing the local conformation. We hypothesize that mutating this residue to glycine (Gly, G)—which has the simplest and most flexible side chain—is likely to disrupt the original rigid constraints. This would introduce moderate conformational flexibility into the catalytic domain. As a result, the enzyme's substrate specificity may be altered, allowing it to bind more efficiently to non‐canonical promoters. This structural alteration may well constitute the key mechanism underlying the elevated activity of the variant. Of note, PEACE 1.0 not only yielded variants with enhanced activity but also placed the key mutations near the catalytic domain rather than in the DNA‑binding domain, which is conventionally targeted for promoter specificity engineering—providing an early indication that the platform can explore unexpected mutational landscapes. These results not only validate the efficacy of the obtained variants in reprogramming transcriptional specificity but also demonstrate that PEACE 1.0 can reliably facilitate continuous in vivo evolution of the GOI.

### 2.5 Optimization of MP to Improve Mutation Efficiency and Diversity

**FIGURE 5:**
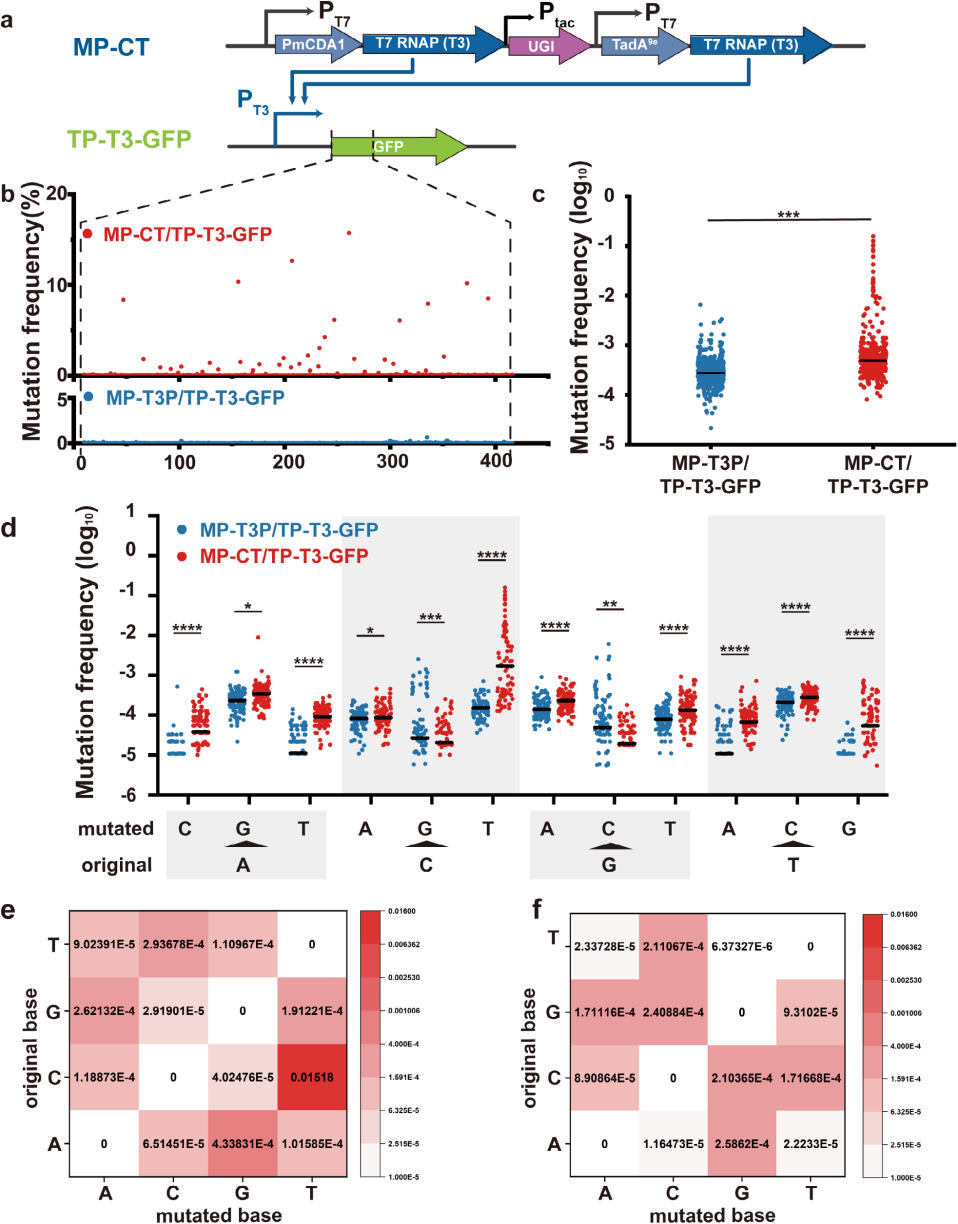
Characterization of the mutagenic activity of MP‐CT. (a) Schematic of the experimental design used to characterize the optimized MP‐CT with eGFP as the reporter. (b) Mutation frequencies at individual sites across the ∼400 bp target gene. Top: MP‐CT (red); Bottom: MP‐T3P (lacking deaminases; blue). (c) Mutation frequency of *E. coli* BL21(DE3)/MP‐T3P/TP‐T3‐GFP and *E. coli* BL21(DE3)/MP‐CT/TP‐T3‐GFP. Mutation frequency is defined as the ratio of mutated reads to the total sequencing depth at a given site. Statistical analysis was performed using one‐way ANOVA; *p* < 0.001 was considered significant. (d) Comparison of mutation frequencies for all possible single‐base substitution types between samples with and without deaminases. (e) Mutational spectrum of MP‐CT/TP‐T3‐GFP. (f) Mutational spectrum of MP‐T3P/TP‐T3‐GFP.

Based on the performance of PEACE 1.0, we targeted the MP for optimization to address its limitations in mutation frequency and diversity. The improvements focused on a two‐pronged strategy: increasing the mutation frequency and expanding the mutational spectrum. To elevate the mutation frequency, the original cytidine deaminase was replaced with the more efficient PmCDA1 [26], and UGI was incorporated to inhibit the BER pathway by inactivating UNG [41]. To counteract the potential mutational bias introduced by repair inhibition and to diversify mutation types, the adenosine deaminase TadA^9e^—selected for its high activity and targeting specificity based on prior studies [22, 35, 42]—was also integrated into the system; TadA^9e^ catalyzes A:T > G:C transitions. Finally, PmCDA1‐T7 RNAP (T3), TadA^9e^‐T7 RNAP (T3), and UGI were co‐assembled into the pCDFDuet‐1, and this optimized mutator element was designated MP‐CT (Figure 5a, Table S2).

To evaluate the mutation efficiency of MP‐CT, *E. coli* BL21(DE3) carrying MP‐CT/TP‐T3‐GFP was subjected to continuous cultivation (12 h per round), with MP‐T3P (without deaminases)/TP‐T3‐GFP serving as the control. Initial KBseq analysis confirmed an intact GOI in the control, whereas MP‐CT induced multiple mutations across the target gene (Figure S5). Subsequent NGS analysis after 60 h of cultivation revealed that MP‐CT efficiently introduced mutations into the GOI, with the highest single‐site mutation frequency reaching 1.57 × 10^−1^ and numerous high‐frequency mutation sites (>1%) distributed broadly and uniformly across the analyzed region (Figure 5b). Boxplot analysis showed that the mutation frequency distribution in the MP‐CT group (3.42 × 10^−4^–7.65 × 10^−4^, with outliers up to 10^−2^–10^−1^) was significantly higher than that in the MP‐T3P control (1.95 × 10^−4^–3.92 × 10^−4^) (Figure 5c). The average mutation frequency of MP‐CT reached 3.49 × 10^−3^, which was 9.2 times that of the control (3.78 × 10^−4^) and 2.3 times that achieved by MP‐AID after 120 h of cultivation (1.54 × 10^−3^), demonstrating faster and more potent mutagenic capability. Furthermore, shortening the cultivation cycle effectively reduced the background mutation frequency (3.78 × 10^−4^ for the 60 h control vs. 1.03 × 10^−3^ for the 120 h MP‐AID control), which is expected to lower the host burden and the false positive rate during evolution.

Analysis of the mutational spectrum indicated that MP‐CT elevated the frequencies of 10 of the 12 base substitution types compared with the control (Figure 5d). Notably, C > T and A > G mutations were predominant, with average frequencies of 1.52 × 10^−2^ and 4.34 × 10^−4^, respectively, substantially exceeding background levels (Figure 5e,f). This confirms that MP‐CT successfully diversifies the mutational spectrum by enhancing C:G > T:A transitions via PmCDA1 and introducing A:T > G:C mutations via TadA^9e^. This richer diversity of mutation types for subsequent in vivo evolution of the GOI makes the system more conducive to meeting diverse evolutionary demands.

### 2.6 Optimization of LP to Improve the Accuracy of Screening

**FIGURE 6:**
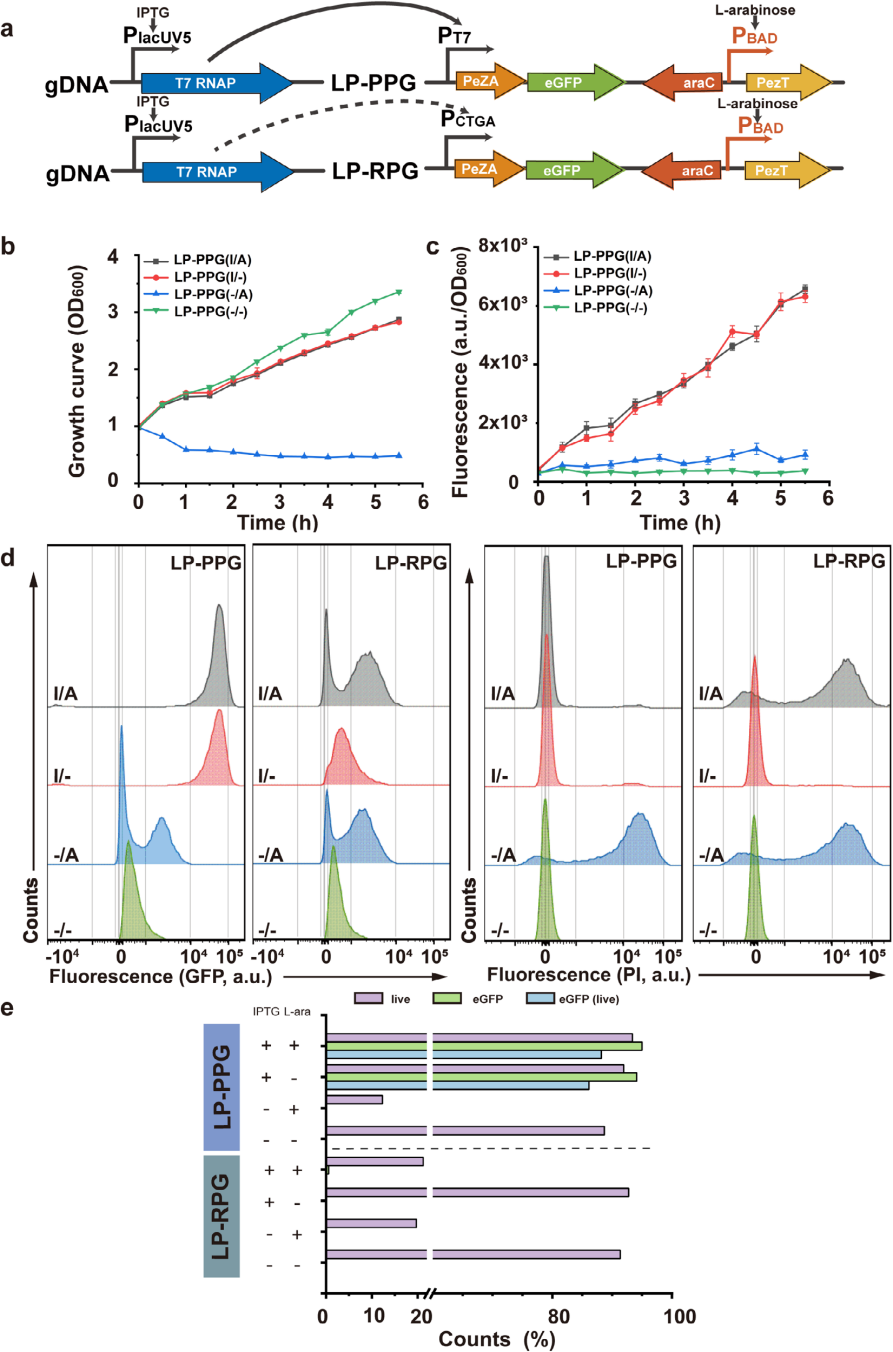
Optimization and verification of the LP. (a) Schematic diagram of the LP optimization strategy. gDNA, genomic DNA. (b) Growth curves of recombinant cells harboring LP‐PPG. "
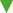
" means no inducer (‐/‐); "
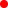
" means the inducer is IPTG (I/‐); "
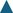
" means *l* ‐arabinose as inducer (‐/A); "
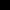
" means IPTG and *l* ‐arabinose as inducers (I/A). (c) Time‐course fluorescence profiles of recombinant cells harboring LP‐PPG. Symbols as defined in (b). (d) FACS analysis of recombinant cells harboring LP‐PPG and LP‐RPG, respectively. The panels show the fluorescence distribution of the cells under different induction conditions. (e) Evaluation of the screening/selection performance of the LP. Based on the FACS data, the proportions of viable cells (live), eGFP‐positive cells (eGFP), and double‐positive cells (live and eGFP) were calculated.

To improve the stringency and efficiency of evolutionary screening/selection, we incorporated eGFP into the LP as a supplementary screening mechanism alongside the TAS. This design enables the TAS to provide continuous selection pressure through toxin–antitoxin interaction during evolution, while eGFP serves as a reporter to enhance screening accuracy through fluorescence quantification. Specifically, we fused the *egfp* gene to the antitoxin *pezA* gene to construct LP‐PPG (Table S2) and LP‐RPG (Table S2), which harbor the *pezA‐egfp* under the control of P_T7_ and P_CTGA_, respectively (Figure 6a). LP‐PPG was employed to assess the functional expression of the PezA‐eGFP fusion protein in the host, whereas LP‐RPG was adapted to monitor the evolutionary trajectory of T7 RNAP.

To evaluate the combined screening/selection effect of the above LP constructs, we transformed LP‐PPG into *E. coli* BL21(DE3) and monitored the growth curves (Figure 6b) and fluorescence intensities (Figure 6c). The results demonstrated that the co‐expression of eGFP and the TAS components exhibited no functional crosstalk, with both systems maintaining their respective activities as designed. To validate FACS as a screening technique for the optimized LP‐PPG, we performed comparative FACS analysis using strains carrying LP‐PPG (positive control, with antitoxin‐eGFP expression) and LP‐RPG (negative control, without antitoxin‐eGFP expression under these conditions). Prior to analysis, cultures were treated with PI to assess cell viability: theoretically, PI stains dead cells red, whereas live cells expressing eGFP emit green fluorescence. As depicted in Figure 6d, the differential fluorescence signals between LP‐PPG and LP‐RPG under varied induction conditions align with the growth disparities previously observed for LP‐PP (Figure 3a) and LP‐RPP (Figure S2). This consistency between FACS results and growth profiles confirms FACS as a reliable screening method for subsequent evolution. Additionally, as shown in Figure 6d, the LP‐PPG group exhibited predominant green fluorescence signals in the 10^4^–10^5^ a.u. range after induction, whereas both the uninduced LP‐PPG and all LP‐RPG groups showed signals primarily within the 0–10^4^ a.u. range. This demonstrates that PezA‐eGFP expression generates fluorescence signals exceeding 10^4 ^a.u., suggesting that during subsequent evolution, cells with signals ≥10^4^ a.u. should be selected as potential positive variants.

To conduct a more in‐depth quantitative analysis of the benefits of synergistic screening using the TAS and eGFP in FACS, we quantified the number of cells in each region of Figure 6d. As shown in Figure 6e, under conditions simulating a successfully evolved host (co‐expression of toxin and antitoxin‐eGFP), the dual‐factor positivity rate (live and eGFP) was approximately 5% lower than the single‐factor rate (live). This marginal difference likely reflects protein expression heterogeneity or natural cell loss during cultivation. Notably, under this condition, the vast majority (>85%) of target cells were correctly identified as dual‐positive, demonstrating the efficacy of FACS in enriching the desired population with minimal loss. In stark contrast, under conditions simulating a non‐evolved host (toxin expression only), the dual‐factor positivity rate was near zero, whereas a substantial fraction (12%–20%) of cells remained viable under the viability‑only criterion. This significant gap indicates that single‐factor screening erroneously counts a substantial number of cells that are viable but do not express antitoxin‐eGFP—cells that may survive transiently through mechanisms such as non‐specific tolerance—as candidate clones. The dual‐factor screening, by requiring both orthogonal signals of host viability and eGFP expression, efficiently excludes such non‑specific survivor clones. This outcome clearly demonstrates that the integration of eGFP substantially enhanced the screening accuracy. Moreover, the throughput capacity of FACS was remarkable, reaching approximately 10^8^ cells per round. This value far surpasses the throughput of plate‐based screening, which typically yields 10^2^–10^4^ colonies per round. This greatly increased throughput, combined with improved screening accuracy, significantly enhanced the overall evolutionary efficiency.

### 2.7 Validation and Application of PEACE 2.0

To comprehensively evaluate the performance of PEACE 2.0 (containing the optimized MP‑CT and LP‑RPG) and to enable a more insightful comparison with PEACE 1.0, we again selected T7 RNAP as the GOI. As in the PEACE 1.0 validation, the objective was to discover variants capable of recognizing the non‐canonical promoter P_CTGA_. Accordingly, the optimized mutator module MP‐CT, the selection module LP‐RPG, and the T7 RNAP expression module TP‐T7P were co‐transformed into *E. coli* BL21(DE3), and the resulting recombinant strain, designated PEACE 2.0‐T7P, was subjected to continuous cultivation and evolution. Following evolution, FACS was used to screen for cells exhibiting fluorescence signals above the predefined threshold of 10^4^ a.u.

**FIGURE 7:**
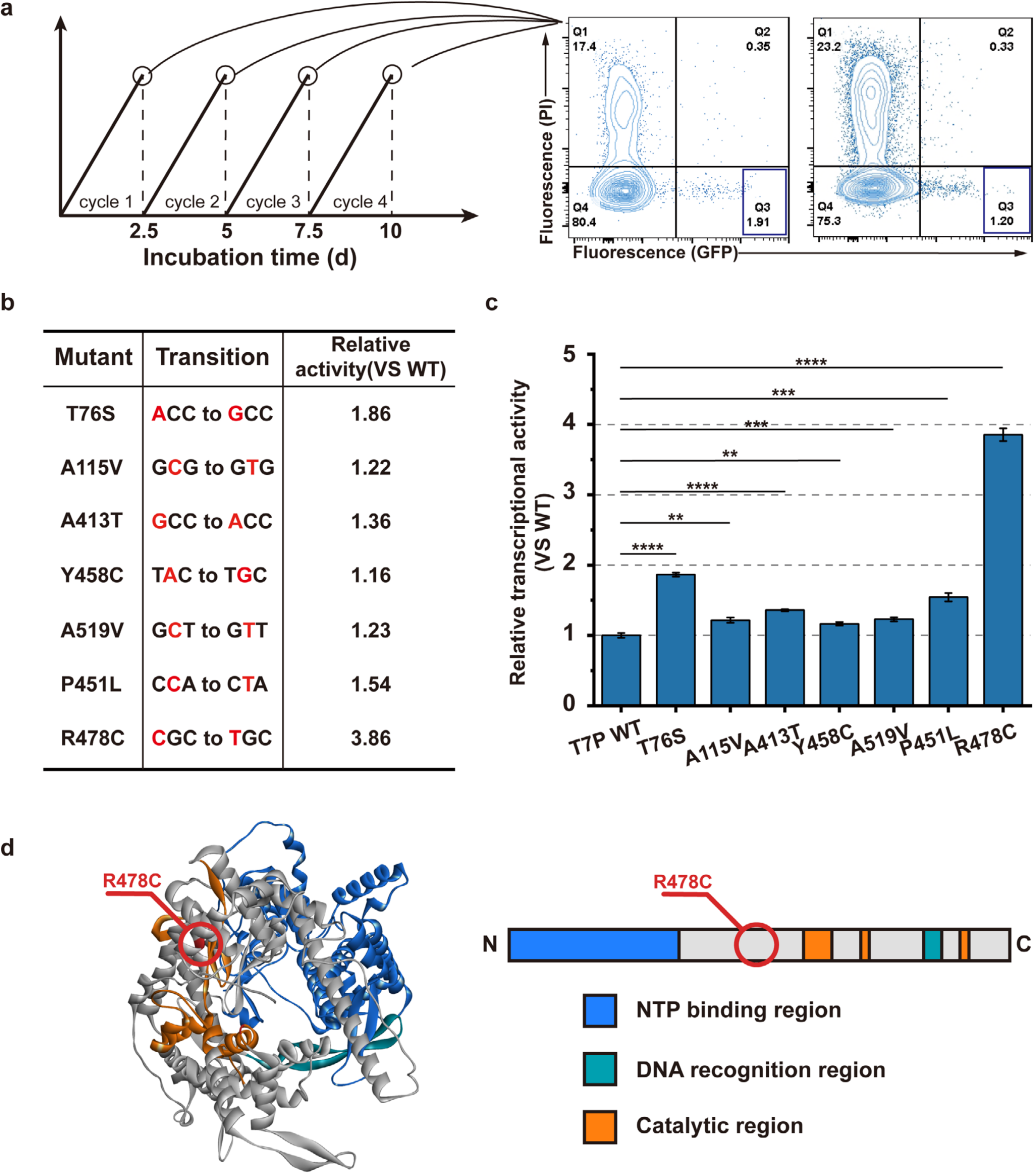
Validation of PEACE 2.0: continuous evolution of T7 RNAP. (a) General scheme of the evolution cycle. (b) Summary of the T7 RNAP variants identified for P_CTGA_. Relative activity is defined as the activity of a variant toward P_CTGA_ relative to that of WT T7 RNAP. Values represent the mean of experiments performed in triplicate. (c) Transcriptional activity of WT T7 RNAP and the selected variants toward P_CTGA_. Activity was measured using *β*‐galactosidase as the reporter. Error bars represent the standard deviations of at least three independent replicates. Statistical significance was assessed by one‑way ANOVA, * indicates *p* < 0.05; ** indicates *p* < 0.01; *** indicates *p* < 0.001; **** indicates *p* < 0.0001. (d) Structural representation of the R478C mutation. The 3D structure (left) and 2D schematic (right) are color‐coded by functional domains: NTP binding domain (blue), DNA binding domain (teal), and catalytic domain (orange); the mutation residue at position 478 is highlighted in red.

In addition to optimizing the functional modules, we refined the evolutionary workflow for PEACE 2.0‐T7P. As shown in Figure 7a, after every five culture cycles, cells emitting green fluorescence ≥10^4^ a.u. were sorted by FACS. These cells were used as the inoculum for the next five evolutionary cycles, and a portion of the sorted sample was simultaneously subjected to KBseq analysis to monitor mutational accumulation. This refinement was intended to accumulate a larger pool of variants, thereby enhancing evolutionary efficiency and increasing the probability of obtaining the desired variants. After 15–20 cycles of evolution, cells exhibiting a fluorescence intensity higher than that of the non‐mutagenized control group were sorted (Figure S6). The sorted clones were then subjected to whole plasmid sequencing using KBseq, yielding a total of seven potential positive mutants (Table S4).

Using *β*‐galactosidase as a reporter, we evaluated the performance of the candidate T7 RNAP variants. As illustrated in Figure 7b,c, variants T76S, A115V, A413T, Y458C, A519V, P451L, and R478C all exhibited elevated transcriptional activity relative to the WT, with the most active variant, R478C, showing an activity 3.86 times that of WT. Structure–activity analysis of this key variant, R478C, revealed a distinct mechanism compared with that of R627G. As shown in Figure 7d, although Arg478 is not located directly within the catalytic domain, it resides in close spatial proximity to it. In the WT structure, the positively charged, long side chain of Arg478 likely anchors and stabilizes the conformation of a nearby structural element critical for catalytic domain integrity. We hypothesize that its mutation to cysteine (C) disrupts this interaction because of the shorter side chain and loss of positive charge. This disruption could induce subtle conformational changes or altered dynamics within the catalytic domain. Such minute structural perturbations may be sufficient to influence the active site geometry, thereby modulating the enzyme's catalytic efficiency through long‐range effects. Consistent with PEACE 1.0, the beneficial mutations obtained with PEACE 2.0 also map near the catalytic domain, further confirming that this platform can systematically discover non‑canonical mutational routes. These results demonstrate that the systematic optimizations in PEACE 2.0 facilitate the generation and identification of improved variants. Compared with PEACE 1.0, the enhanced system enriches a broader pool of functional target protein mutants and achieves this with higher fidelity and in a significantly shorter evolution time. This underscores that PEACE 2.0 provides a more efficient and robust platform for continuous in vivo protein evolution.

### 2.8 The Continuous Evolution of Transcription Factor PsiR

After validating PEACE 2.0 through the evolution of T7 RNAP, we extended the application of this system to re‐engineer the ligand specificity of the transcription factor (TF) PsiR. PsiR binds to P*_psiA_* promoter and inhibits the transcription of genes under its control [43]. In the presence of *d*‐allulose, PsiR dissociates from the promoter and relieves transcriptional inhibition. Originally, PsiR was used to specifically sense endogenous *d*‐allulose in the host and served as a key regulatory element in the development of enzyme variants for high‐yield *d*‐allulose biosynthesis. During continuous evolution, we therefore selected *d*‐tagatose, *d*‐fructose, and *d*‐mannose as inducers, with the aim of obtaining PsiR variants that can distinctly respond to each of these respective monosaccharides. Such variants would provide significant application value for the development of various monosaccharide biosensors, promoting efficient production of high‐value sugars, optimizing raw material utilization, and advancing industrial development.

**FIGURE 8:**
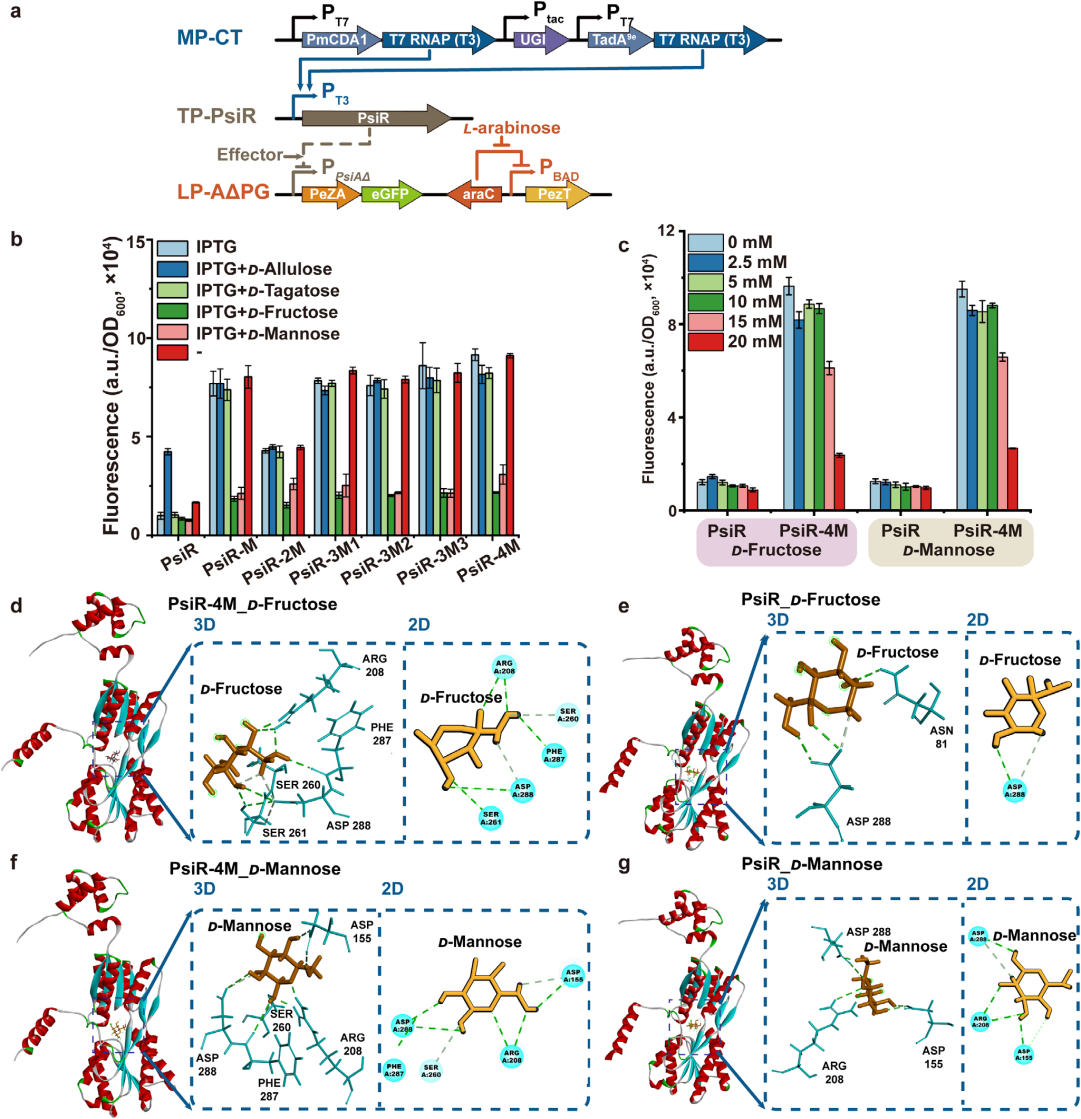
Evolution of PsiR using the PEACE 2.0. (a) Schematic of the PEACE 2.0 continuous evolution strategy targeting the transcription factor PsiR. (b) Transcriptional activity profiles of PsiR and its variants under different induction conditions, measured as the fluorescence intensity of the reporter protein. Values represent means from three independent replicates. (c) Response sensitivity of PsiR‐4M to *d*‐fructose and *d*‐mannose. Data points represent means from triplicate experiments. (d–g) Molecular docking analysis of: PsiR‐4M variant with *d*‐fructose (d), WT PsiR with *d*‐fructose (e), PsiR‐4M variant with *d*‐mannose (f), and WT PsiR with *d*‐mannose (g).

First, we replaced the P_T7_ promoter driving *pezA‐egfp* in LP‐PPG with the P*_psiAΔ_* promoter, thereby constructing LP‐AΔPG (Table S2), a plasmid tailored specifically for the evolution of PsiR. To verify the response of WT PsiR to the P*_psiAΔ_*, we monitored the growth trajectories of cells carrying both TP‐T7‐PsiR and LP‐AΔPG within 5.5 h post‑induction under our experimental conditions (Figure S7). The results revealed that WT PsiR exhibited reduced transcriptional activation of PezA in the presence of *d*‐allulose when regulated by P*_psiAΔ_*, in contrast to the previously used P_T7_. This low background activity allows more pronounced observation of signal changes in positive variants during continuous evolution, thereby enhancing screening/selection efficiency. Next, the PsiR expression vector TP‐PsiR (driven by P_T3_, Table S2), LP‐AΔPG, and MP‐CT were co‐transformed into *E. coli* BL21(DE3) to construct the recombinant strain. The complete in vivo continuous evolution process was designated PEACE 2.0‐PsiR, and its schematic illustration is presented in Figure 8a.

Following continuous growth coupled with selection, the evolved populations were analyzed by FACS, and cells exhibiting fluorescence intensities different from those of the non‐mutagenized control group were sorted (Figure S8a). Each sorted strain, along with the non‐evolved WT control, was individually induced and cultured in the presence of *d*‐allulose, *d*‐tagatose, *d*‐fructose, *d*‐mannose, and a sugar‐free control. Subsequently, the fluorescence values of the cultures were measured (Figure S8b). Based on these comparative data, Sanger sequencing was performed on the GOI from all strains displaying fluorescence values distinct from those of the non‐mutagenized control (Table S5). Using Emerald green fluorescent protein as a reporter, we evaluated the regulatory properties of the selected PsiR variants obtained from continuous evolution in the presence of various inducers (Figure 8b). In contrast to the WT, variants PsiR‐M (G285D), PsiR‐2M (G285D/C286Y), PsiR‐3M1 (G285D/C286Y/F315F), PsiR‐3M2 (G285D/C286Y/E316K), PsiR‐3M3 (G285D/C286Y/E309E), and PsiR‐4M (G285D/C286Y/F292L/R322R) exhibited a fundamental alteration in regulatory logic: they no longer responded to activation by *d*‐allulose, but instead were specifically repressed by *d*‐fructose and *d*‐mannose. These findings indicate that the regulatory mechanism of PsiR was successfully switched from a *d*‐allulose‐dependent activation mode to a *d*‐fructose/*d*‐mannose‑dependent repression mode. As shown in Figure 8c, the variants consistently exhibited insensitivity to *d*‐fructose and *d*‐mannose at concentrations below 15 mm, whereas a significant transcriptional inhibitory effect was observed at concentrations exceeding 15 mm, further substantiating their repressive regulatory phenotype.

To gain preliminary insights into the potential molecular basis for the altered ligand specificity of the evolved PsiR variants, we predicted the three‐dimensional structures of both WT PsiR and the PsiR‐4M variant using AlphaFold3. Subsequently, molecular docking was performed with AutoDock Vina [44] to explore their potential interactions with *d*‐fructose and *d*‐mannose, and the lowest energy binding conformations were selected for further analysis. Examination of the lowest energy docking poses suggested that both ligands could bind to the active pocket of the 4M variant, with binding energies of −18.5 and −15.4 kcal/mol. Analysis of potential hydrogen bond interactions indicated that in the 4M variant, *d*‐fructose could form a hydrogen bond network with residues including Arg208, Ser260, Ser261, Phe287, and Asp288 (Figure 8d), whereas in the WT, interactions were primarily with Asn81 and Asp288 (Figure 8e). Similarly, *d*‐mannose showed potential for interactions with Asp155, Arg208, Ser260, Phe287, and Asp288 in the variant (Figure 8f), compared with a more limited set of residues (Asp155, Arg208, Asp288) in the WT (Figure 8g). Collectively, these computational analyses provide a tentative structural hypothesis: the mutations in PsiR‐4M, although not directly involved in ligand binding, may enhance affinity by inducing local conformational adjustments that optimize the hydrogen bond network. This in silico analysis serves to offer a rational model for the observed phenotype and to lay the groundwork for future mechanistic studies.

Many established in vivo continuous evolution systems have primarily targeted antibiotic resistance proteins or other directly selectable traits. Although this approach is convenient, it inherently limits the broader applicability of continuous evolution, as expanding its scope often depends on the development of suitable biosensors. To address this challenge, we selected PsiR—a transcription factor responsive to a rare sugar—as a model target for evolution by PEACE 2.0. The successful reprogramming of PsiR ligand specificity not only adds a valuable case to the repertoire of continuously evolved proteins but also demonstrates a more general principle: the ability to engineer transcription factors using PEACE provides a pathway to generate customized biosensors. These biosensors can then, in principle, serve as selection modules for evolving downstream enzymes, thereby extending the reach of continuous evolution to a wider range of proteins beyond those directly coupled to selection. In this regard, our work expands both the scope and the utility of in vivo continuous evolution, offering a practical strategy for synthetic biology and metabolic engineering applications.

## 3 Discussion

The PEACE system was developed to overcome the key bottleneck of manual intervention in traditional directed evolution by establishing a streamlined, growth‐coupled continuous evolution framework in *E. coli*. Its core design integrates two orthogonal yet coupled processes: targeted in vivo mutagenesis and function‐dependent cell survival.

MP exploits the high‐fidelity transcription machinery of T7 RNAP coupled with a BD to achieve localized mutagenesis [35]. We selected the T7 RNAP (T3) variant fused to a BD to ensure orthogonal transcription from the P_T3_ on the target plasmid, thereby focusing mutations exclusively on the GOI without interfering with the system's own expression circuitry [34]. This strategy provides a wider and more processive editing window compared with Cas9‐dependent base editors, which is advantageous for in vivo continuous evolution. A critical mechanistic feature of the initial system (PEACE 1.0) was the deliberate retention of the host's native BER pathway. The cytidine deaminase AID catalyzes C > U deamination on transiently single‐stranded DNA [45]; the resulting U:G mismatch is then processed by BER, which can occasionally introduce mutations during resynthesis [46, 47]. This choice, while resulting in a lower overall mutation frequency, was designed to harness the mutational diversity inherently generated by the repair process itself. The recovery of gain‐of‐function T7 RNAP variants carrying transversion mutations (Table  S4) validates this design rationale, confirming that beneficial mutations can arise from the stochastic outcomes of BER, not solely from direct C > T transitions. Thus, the mutation frequency of PEACE 1.0 is modest. Nevertheless, it remains significantly higher than that of the background control. Importantly, it successfully achieves the intended goal of broadening the mutational spectrum. This design strategy informed the optimization of PEACE 2.0 by providing a clear rationale for two key objectives: increasing mutation frequency while preserving mutational spectrum as much as possible.

The optimization from PEACE 1.0 to 2.0 strategically rebalanced the trade‐off between diversity and efficiency in the MP. By employing a dual‐deaminase strategy—combining the efficient PmCDA1 (with UGI) to elevate the C:G > T:A frequency [47, 48] and integrating TadA^9e^ to ensure A:T > G:C mutations [35, 49, 50]—the system achieves high‐frequency mutagenesis without sacrificing a broad mutational spectrum. This refined module enables an average mutation frequency of 3.49 × 10^−3^ per bp within a shortened 60‐h evolution cycle. The increased efficiency directly reduces the required evolution time, which in turn lowers the background mutation accumulation (to 3.78 × 10^−4^), thereby minimizing host burden and spurious mutations in system components. In comparison with related systems, PEACE 2.0 achieves a mutagenic rate comparable to that of the optimized eMutaT7^transition^ (∼5.3 × 10^−3^ per bp, 80 h) but within a condensed timeframe [29]. Its performance substantially accelerates the classic MutaT7 timeline (6 × 10^−3 ^per bp, 15 days) [27]. Furthermore, unlike systems that require separate handling for different mutation types, the integrated, concurrent expression of both deaminases from a single plasmid in PEACE 2.0 offers a streamlined and stable platform for maintaining diverse mutant libraries under continuous selection. Recent advances in adenosine deaminase engineering further illuminate paths for refining the PEACE platform. For instance, TadA orthologs capable of simultaneous cytosine and adenine editing with markedly reduced RNA off‐target activity have been recently developed [51]. In parallel, studies dissecting how distinct TadA variants influence DNA sequence recognition have provided valuable guidance for optimizing DNA substrate editing [52]. These findings offer actionable insights for the ongoing and future optimization of the PEACE system, some of which are already being implemented.

In vivo continuous evolution requires a selection pressure that is stringently coupled to the desired protein function, sustainable over long periods, and minimally burdensome to host metabolism. Although antibiotic resistance is commonly used, it can trigger non‐specific stress responses and genetic instability [53, 54, 55, 56]. To circumvent these issues, we employed a TAS‐based strategy. The selected PezT/PezA pair [38], a type II TAS, provides a robust and orthogonal logic gate: cell survival is strictly contingent upon the expression of the antitoxin, which in turn is linked to the activity of the evolved protein. This protein–protein interaction mechanism, which is self‑contained, offers direct and reversible growth control that is ideal for long‐term cultivation without external additives. In PEACE 2.0, this core logic was enhanced to address a fundamental challenge in growth‐coupled selection: non‑specific host tolerance that gives rise to undesired surviving clones. We introduced an orthogonal verification layer by fusing the antitoxin to eGFP, creating a dual‐factor screening gate that requires both cell viability (via the TAS) and a target function signal (via fluorescence). This design removed a substantial non‑specific survivor population accounting for 12%–20% of total cells and enabled ultrahigh‐throughput sorting via FACS. It exemplifies a key principle of the PEACE system: increasing the number of orthogonal screening dimensions significantly improves the precision and efficiency of the evolutionary trajectory.

T7 RNAP was selected as a model GOI primarily to validate the operational feasibility of PEACE, using the well‐defined challenge of reprogramming its promoter specificity as a test case. Its high specificity for P_T7_ and extremely low basal activity toward the heterologous P_CTGA_ (>100‐fold lower) established a clear functional starting point and a stringent evolutionary challenge [34, 57, 58, 59]. PEACE successfully overcame this hurdle, evolving variants with up to 8.31 times the activity of WT on P_CTGA_. Notably, the most effective mutations identified in our study, such as R627G and R478C, are located near the catalytic domain, in contrast with prior strategies that focused on engineering the DNA‐binding domain directly [34]. This suggests that PEACE explored an alternative mutational path, potentially enhancing promoter engagement through allosteric effects on catalytic efficiency or global polymerase dynamics, rather than by directly altering binding interface residues. These results demonstrate that the PEACE system is effective for continuous evolution and is capable of exploring a broad mutational landscape to identify functional solutions. The successful redirection of T7 RNAP specificity, achieved through distal mutations that influence enzyme dynamics, highlights the platform's utility for advancing protein engineering goals where rational design is challenging.

To further demonstrate the capability of PEACE 2.0 for reprogramming complex protein functions, we applied it to evolve the ligand specificity of the transcription factor PsiR. The system successfully converted this *d*‐allulose‐responsive activator into a variant (PsiR‐4M) that acts as a repressor in response to *d*‐fructose and *d*‐mannose, achieving a functional switch not previously achieved through continuous evolution. The molecular basis for this reprogramming was explored by computational analysis. Docking simulations suggested enhanced binding affinity for the new ligands, and structural modeling indicated that the acquired mutations likely act synergistically to remodel the binding pocket through a combination of altered electrostatics, flexibility, and stability. Although detailed mechanistic validation awaits further study, these predictions provide a testable hypothesis and a clear starting point for such investigations. This work establishes a practical in vivo evolution–computational validation pipeline for engineering TFs with desired specificities, even in the absence of extensive structural data. The evolved PsiR‐4M variant, which maintains orthogonality in *E. coli*, serves as both a functional biosensor component and a case study showcasing how PEACE can be deployed to expand the toolbox of synthetic biology.

Overall, PEACE establishes a continuous evolution framework within *E. coli* that integrates two synergistic components: targeted in vivo mutagenesis and stringent growth‐coupled selection. This integration simplifies the experimental workflow by relying on a single microbial host and combining mutation and selection into a continuous process. The system's core design, which couples a protein's functional output directly to cell survival via a TAS logic gate, provides a generalizable strategy for maintaining continuous evolution. The addition of fluorescence‐based sorting further enhances the fidelity of variant recovery.

Methodologically, PEACE provides a rigorous and generalizable framework for directing the evolution of diverse proteins, as exemplified by the reprogramming of T7 RNAP promoter specificity and the switching of PsiR ligand response. These results validate the utility of this framework for precise protein engineering tasks. However, the broader potential of PEACE lies in its extensible “biosensor‑coupled evolution” strategy: as demonstrated in this work, transcription factors such as PsiR can be rapidly evolved into customized biosensors, which in turn serve as selection modules for evolving downstream metabolic enzymes that are otherwise difficult to couple directly to a selective pressure. This strategy directly bridges the research gap of “expanding the application scope” raised in the Introduction by providing a feasible technological pathway. Thus, PEACE is an efficient platform for engineering transcriptional regulators. More broadly, it serves as a generalizable tool for metabolic engineering and synthetic biology. By constructing biosensors on demand, the system can be adapted to a broad range of target proteins, provided that their activity can be linked to a detectable metabolic signal. In this way, it extends continuous evolution to a wide variety of proteins. Future efforts will focus on applying this strategy to optimize practical metabolic pathways and further simplify biosensor construction workflows, thereby enabling broader biotechnological applications.

## 4 Conclusion

In conclusion, this study has developed an in vivo continuous evolution system, PEACE, whose feasibility and efficacy have been validated through the successful continuous evolution of both T7 RNAP and PsiR. PEACE offers a versatile and efficient platform for optimizing protein functions and holds promise for applications in protein engineering, metabolic engineering, and synthetic biology. The demonstrated performance of PEACE not only provides a practical foundation for future protein evolution efforts but also expands the available toolkit for addressing complex biological engineering challenges.

## 5 Methods

### 5.1 Bacterial Strains, Media and Growth Conditions

The strains used in this study are listed in Table S1. All *E. coli* strains were cultured in Luria‐Bertani (LB) medium at 37°C and 200 rpm. The cultivation strategy for continuous evolution of *E. coli* BL21(DE3) was as follows: (i) PEACE 1.0 was performed in Terrific Broth (TB) medium at 25°C and 200 rpm; (ii) PEACE 2.0 was performed in TB medium at 25°C and 700 rpm. The plasmids and primers used in this study are listed in Tables S2 and S3, respectively.

### 5.2 Construction of MP and TP

The vector pCDFDuet‐1 was used as the backbone for construction of the MP. The gene encoding the AID‐T7 RNAP (T3) fusion protein [34] was synthesized (GENEWIZ Inc., Suzhou, Jiangsu, China) and inserted downstream of the first P_T7_ of pCDFDuet‐1 via *NcoI/XhoI* digestion and ligation; the resulting plasmid was designated MP‐AID. Genes encoding the PmCDA1‐linker [26] and TadA^9e^‐linker [35] fusions were synthesized separately (GENEWIZ Inc., Suzhou, Jiangsu, China). The aid on MP‐AID was replaced with *Pmcda1‐linker* and *tadA^9e^‐linker* by *NcoI/HindIII* digestion and ligation, yielding MP‐CL and MP‐TL, respectively. Using primers CTL OS‐F/CTL OS‐R (Table S3) and CL OS‐F/CL OS‐R (Table S3), the expression cassette for the *tadA^9e^‐linker‐T7 rnap (T3)* fusion from MP‐TL was assembled with the expression cassette for the *Pmcda1‐linker‐T7 rnap (T3)* fusion and the T7 terminator from MP‐CL by one‐step cloning. The expression cassette of the uracil‐DNA glycosylase inhibitor gene (*ugi*) [26] (GENEWIZ Inc., Suzhou, Jiangsu, China) was inserted between the two aforementioned fusion protein cassettes, yielding the final construct MP‐CT.

Meanwhile, the target gene encoding WT T7 RNAP was inserted into pET20b using primers T7P‐F and T7P‐R by megaprimer PCR of the whole plasmid (MEGAWHOP) [60]. The P_T7_ promoter (5′‐TAATACGACTCACTATA‐3′) on the resulting plasmid was then replaced with P_T3_ (5′‐TAATAACCCTCACTATA‐3′) using primers P_T3_ SDM‐F/P_T3_ SDM‐R (Table S3) by site‐directed mutagenesis (SDM); this construct was designated TP‐T7P. Similarly, the gene encoding the transcription factor PsiR (WT) was used as the template, and the *T7 rnap* on TP‐T7P was replaced with the *psiR* using primers PsiR‐F/PsiR‐R (Table S3) by MEGAWHOP, resulting in the construct TP‐PsiR.

### 5.3 In Vivo Continuous Mutagenesis of eGFP

The recombinant plasmid pET24a‐*egfp* was constructed as TP‐T7‐GFP, and its P_T7_ was mutated to P_T3_ by SDM to generate TP‐T3‐GFP. Recombinant *E. coli* BL21(DE3) strains harboring TP‐T7‐GFP, TP‐T3‐GFP, MP/TP‐T7‐GFP, and MP/TP‐T3‐GFP were grown overnight in LB medium containing 100 µg/mL ampicillin and 50 µg/mL streptomycin, respectively.

The overnight cultures were subcultured at a 1:10 dilution into TB medium supplemented with the appropriate antibiotics and 0.4 mm isopropyl *β*‐*d*‐thiogalactoside (IPTG) for continuous cultivation. During the PEACE 1.0 evolution process, continuous culture was conducted in 100 mL conical flasks. Each round lasted 24 h, and a total of five rounds were performed (25°C, 200 rpm). During the PEACE 2.0 evolution process, continuous culture was conducted in 24 deep‐well plates. Each round lasted 12 h, and a total of five rounds were performed (25°C, 700 rpm).

The cultured samples were then analyzed by FACS as follows: a total of 0.5 mL of cell culture was washed with PBS buffer (10 mm, pH 7.4) (4°C, 5000 g, 5 min) and re‐suspended in 4 mL of PBS buffer. The optical density at 600 nm (OD_600_) of the cell suspension was measured, and the suspension was appropriately diluted. Propidium iodide (PI, 1 mg/mL) was added at 0.5 µL per 1 mL of diluted sample to distinguish dead cells via fluorescence detection. A total of 600 µL of the stained dilution was sorted using a BD FACSAria III Cell Sorter (Becton, CA, USA; excitation/emission wavelength 488/561 nm). Data were collected and analyzed using FlowJo v10 software.

### 5.4 Next‐Generation Sequencing (NGS)

NGS was performed to measure the mutation frequency. Sample preparation was as follows: the sorted recombinant *E. coli* BL21(DE3)/MP/TP‐T3‐GFP and *E. coli* BL21(DE3)/TP‐T3‐GFP cells were incubated at 37°C and 200 rpm for 4–6 h, after which their plasmid mixtures were separately extracted using a SanPrep Column DNA gel extraction kit (Sangon Biotech Co., Ltd., Shanghai, China). A 426 bp fragment of the *egfp* was then amplified from the plasmid mixtures using primers GNGS‐F/GNGS‐R (Table S3). The PCR products were sent to Sangon Biotech Co., Ltd. for NGS, and the mutation diversity and frequency of *egfp* were analyzed according to the report provided by the company. Mutation frequency was defined as the ratio of mutated reads to total reads at each base position.

### 5.5 Construction of LP

The genes encoding the following toxin‐antitoxin pairs—CcdB/CcdA [37], PezT/PezA [38], SezT/SezA [39], and Zeta toxin/Epsilon [40]—were synthesized. The vector pRSFDuet‐1 was used as the backbone for expression of these proteins. The genes encoding the toxins CcdB, PezT, SezT, and Zeta toxin were each inserted downstream of the second P_T7_ of pRSFDuet‐1, and the genes encoding the antitoxins CcdA, PezA, SezA, and Epsilon were each inserted downstream of the first P_T7_ (GENEWIZ Inc., Suzhou, Jiangsu, China). Subsequently, the second P_T7_ in each of these recombinant plasmids was replaced with P_BAD_ from pBAD/His by MEGAWHOP. At the same time, the *araC* from pBAD/His was inserted into the above recombinant plasmids by MEGAWHOP using primers araBAD‐F araBAD‐R (Table S3), yielding LP‐CC (Table S2), LP‐PP, LP‐SS (Table S2), and LP‐EZ (Table S2), respectively.

The first P_T7_ of LP‐PP was replaced with P_CTGA_ (5′‐TAATACCTGACACTATA‐3′) using primers P_CTGA_SDM‐F/P_CTGA_SDM‐R (Table S3) to generate LP‐RPP. Using primers LG‐F/LG‐R (Table S3), the gene encoding a linker‐eGFP fusion was inserted downstream of PezA in LP‐PP and LP‐RPP by MEGAWHOP, obtaining the recombinant plasmids LP‐PPG and LP‐RPG. Using primers PA‐F/PA‐R (Table S3), the P*_psiAΔ_* was introduced by MEGAWHOP to replace the P_T7_ on LP‐PPG, yielding LP‐AΔPG.

### 5.6 Growth Curve Assays

LP‐CC, LP‐PP, LP‐SS, and LP‐EZ were separately transformed into *E. coli* BL21(DE3). The resulting recombinant strains were inoculated into 10 mL of LB medium (30 µg/mL kanamycin) and incubated overnight at 37°C and 200 rpm. The cultures were then transferred to 50 mL of fresh LB medium (30 µg/mL kanamycin) at a 1:20 ratio and incubated (37°C, 200 rpm) until the OD_600_ reached 0.6. After addition of inducers (0.4 mm IPTG and 0.3% *l*‐arabinose), the cultures were incubated at 25°C and 200 rpm for 5.5 h, and the OD_600_ values were measured at 0.5 h intervals. Control groups with only IPTG, only *l*‐arabinose, and without any inducer were processed in parallel.

To monitor the growth curve of LP‐PPG under different induction conditions (IPTG/*l*‐arabinose, IPTG alone, *l*‐arabinose alone, no inducer), the OD_600_ of samples collected every 0.5 h was measured. Simultaneously, an appropriate amount of bacterial suspension was taken, washed with PBS buffer (10 mm, pH 7.4), and resuspended. A 200 µL aliquot of the resuspension was transferred to a transparent 96‐well plate and a black 96‐well plate. Absorbance at 600 nm was measured in the transparent plate using a Spark Multimode Microplate Reader (Tecan, Switzerland), and fluorescence (excitation/emission 488/561 nm) was measured in the black plate using the same instrument.

### 5.7 SYTO9/PI Staining Assay

A SYTO9/propidium iodide (PI) staining assay was used to evaluate the lethal effect of toxin–antitoxin systems (TAS) on *E. coli* [61]. A 1 mL aliquot of the above culture was centrifuged (4°C, 5000 g, 10 min). The cell pellet was washed with 0.9% NaCl solution and finally resuspended in 1 mL of 0.9% NaCl solution. A 950 µL aliquot of the cell suspension was mixed with 0.50 µm SYTO9 and 45 µm PI (Invitrogen, Carlsbad, CA, USA). After incubation for 15 min in the dark, 5 µL of the mixture was spread onto a glass slide and sealed with clear nail polish. The sample was then observed and photographed using a TCS SP8 STED fluorescence microscope (Leica Microsystems, Wetzlar, Germany; excitation/emission 488/552 nm).

### 5.8 Optimization and Screening/Selection Efficiency Analysis of LP

The LP‐PPG and LP‐RPG constructs were individually transformed into *E. coli* BL21(DE3). The resulting recombinant strains were each inoculated into 10 mL LB medium (30 µg/mL kanamycin) and incubated overnight at 37°C with shaking at 200 rpm. The cultures were then transferred to 50 mL of fresh LB medium (30 µg/mL kanamycin) at a 1:20 ratio and incubated (37°C, 200 rpm) until the OD_600_ reached 0.4. Inducers (0.4 mm IPTG and 0.3% *l*‐arabinose) were added, and the cells were incubated for 12 h at 25°C with shaking at 200 rpm. Control groups with only IPTG, only *l*‐arabinose, and no inducer were processed in parallel. FACS analysis was performed on the cultures as described above, and the number of cells exhibiting the corresponding fluorescence in each gate was counted.

### 5.9 In Vivo Continuous Evolution of T7 RNAP

For PEACE 1.0, MP‐AID, TP‐T7 RNAP, and LP‐RPP were sequentially transformed into *E. coli* BL21(DE3). The recombinant strain was incubated in LB medium containing 30 µg/mL kanamycin, 100 µg/mL ampicillin, and 50 µg/mL streptomycin at 37°C and 200 rpm for 12 h. The culture was then diluted 1:10 into TB medium with the respective antibiotics and incubated for 1.5 h under the same conditions. After addition of 0.4 mm IPTG, the culture was incubated at 25°C and 200 rpm for 1 h, after which 0.3% *l*‐arabinose was added and incubation continued for 24 h. The culture was then diluted 1:10 into fresh TB medium with the respective antibiotics to initiate the second cycle. After a total of five cycles, the culture was diluted into fresh LB medium containing the corresponding antibiotics, 0.4 mm IPTG, and 0.3% *l*‐arabinose and incubated under the same conditions for 5.5 h. The cultured product was then spread onto LB agar plates supplemented with 100 µg/mL ampicillin and 0.3% *l*‐arabinose and incubated overnight at 37°C. The sequences of the T7 RNAP variants were confirmed by KBseq and Sanger sequencing (Sangon Biotech Co., Ltd., Shanghai, China).

For PEACE 2.0, MP‐CT, TP‐T7P, and LP‐RPG were successively transformed into *E. coli* BL21(DE3). After culturing for 12 h in LB medium using the same strategy as above, the culture was transferred at a 1:10 ratio to TB medium containing the corresponding antibiotics and IPTG in a 24 deep‐well plate. After every five cycles of cultivation (12 h per round, with *l*‐arabinose added from the second round onward), single cells exhibiting fluorescence values higher than those of the LP‐RPG control were sorted by FACS using the same method as described in Section 5.3. The sorted cells were then inoculated into fresh LB medium and cultured for 8 h to reach the stationary phase, and evolution was continued until the next five‐cycle block was completed. Subsequently, KBseq and Sanger sequencing (Sangon Biotech Co., Ltd., Shanghai, China) were performed on the culture broth after the aforementioned cultivation.

### 5.10 In Vivo Characterization of T7 RNAP Variants

The genes encoding the selected T7 RNAP variants were individually cloned into pET24a to generate the corresponding recombinant plasmids. In addition, the *bgaD* gene encoding *Bacillus circulans* ATCC 31382 *β*‐galactosidase [62] was cloned downstream of the first P_T7_ of pCDFDuet‐1, and this P_T7_ was subsequently replaced with P_CTGA_ to generate pCDFDuet‐P_CTGA_‐*gal*.

Each recombinant plasmid encoding a T7 RNAP variant was co‐transformed with pCDFDuet‐P_CTGA_‐*gal* into the *E. coli* BL21(DE3)‐Test strain. After cultivation of the recombinant cells, *β*‐galactosidase activity was measured as follows: 100 µL of crude enzyme solution obtained by sonication of the cells was added to 1.8 mL of PBS buffer (50 mm, pH 5.5) and pre‐warmed at 50°C for 5 min; then, 100 µL of substrate solution (20 mm *o*‐nitrophenyl‐*β*‐*d*‐galactopyranoside, *o*NPG) was added and mixed. After a 10 min reaction at 50°C, 1 mL of 1 m Na_2_CO_3_ was immediately added to stop the reaction, and the absorbance at 420 nm was measured using a UV–vis spectrophotometer (UV‐1800, JIAPENG Analytical Instrument Co., Ltd., Shanghai, China). Under the conditions described above, one unit (U) of β‐galactosidase activity was defined as the amount of enzyme that catalyzes the hydrolysis of *o*NPG to produce 1 µmol *o*‐nitrophenol per minute [63].

### 5.11 In Vivo Continuous Evolution of PsiR

MP‐CT, TP‐PsiR, and LP‐AΔPG were transformed into *E. coli* BL21(DE3). Following the same strategy as above, the cultures were grown in LB medium for 12 h and then subcultured at a 1:10 ratio into TB medium containing the corresponding antibiotics and IPTG in 24 deep‐well plates. After five rounds of cultivation (12 h per round, with *l*‐arabinose added from the second round onward), single cells exhibiting fluorescence values higher than those of the LP‐RPG control were sorted by FACS. The sorted cells were plated onto fresh LB solid medium and cultured for 8 h. Subsequently, all single colonies from the plate were picked and inoculated into LB medium containing the corresponding antibiotics and IPTG, and cultured at 37°C and 700 rpm for 8 h. Five aliquots of the resulting culture were inoculated into TB medium containing the corresponding antibiotics and IPTG. After incubation at 37°C and 700 rpm for 1.5 h, four aliquots were individually supplemented with *d*‐tagatose, *d*‐fructose, or *d*‐mannose, while the fifth aliquot remained without any inducer. All aliquots were further incubated at 37°C and 700 rpm for 24 h. The cultures were then washed and resuspended in PBS buffer (10 mm, pH 7.4). The optical density at 600 nm (OD_600_) and the fluorescence (excitation/emission 488/561 nm) were measured using a Spark Multimode Microplate Reader (Tecan, Switzerland). Bacterial cultures exhibiting fluorescence profiles distinct from that of the WT group were subjected to Sanger sequencing (GENEWIZ Inc., Suzhou, Jiangsu, China).

### 5.12 In Vivo Characterization of PsiR Variants

The genes encoding the selected PsiR variants were individually cloned into pSB1C3‐PsiR‐P*_psiAΔ_*‐*memerald*, replacing the original *psiR* (WT) in the plasmid, to obtain the validation plasmids for the different variants. Each plasmid was transformed into *E. coli* BL21(DE3). Fluorescence values were measured for the recombinant bacteria cultured at 37°C and 700 rpm for 12 h under different induction conditions (addition of *d*‐allulose, *d*‐tagatose, *d*‐fructose, *d*‐mannose, or no inducer).

Subsequently, using the same validation strains and culture strategy, the fluorescence values of PsiR and PsiR‐4M were measured at different *d*‐fructose induction concentrations (0, 2.5, 5, 10, 15, and 20 mm). Similarly, the fluorescence values of PsiR and PsiR‐4M were measured at different *d*‐mannose induction concentrations (0, 2.5, 5, 10, 15, and 20 mm).

### 5.13 Quantification and Statistical Analysis

Statistical significance was assessed by comparing the mean values (±S.D.) using one‐way ANOVA; *p*‐values < 0.01 were considered significant (*p < 0.05, **p < 0.01, ***p < 0.001, ****p < 0.0001).

## Author Contributions

X.Y.Z., Z.Z.L., Y.X., M.J.S., G.Z.Z., and Z.G.L. conducted experiments. Z.Z.L., J.W., and X.Y.Z. conceived and designed research. Z.Z.L. and J.W. applied for the supporting funding. X.Y.Z., Z.Z.L., D.M.R., X.Q.C., S.W., and Y.H. analyzed data. X.Y.Z. and Z.Z.L. wrote the manuscript. Z.Z.L. and J.W. polished the manuscript. All authors read and approved the manuscript.

## Funding

This work was supported by the National Natural Science Foundation of China (NSFC) [32430081 and 22578168] and the Jiangsu Provincial Key Research and Development Program (Modern Agriculture) [No. BF2025319].

## Conflicts of Interest

The authors declare no conflicts of interest.

## Supporting information




**Supporting File**: advs76924‐sup‐0001‐SuppMat.docx.

## Data Availability

All data are provided in the included main text and supporting figures and tables. Raw data of NGS for mutation rate have been deposited to the BioProject/SRA, with Accession No. BioProject: PRJNA902431 and No. BioProject: PRJNA1199376. Micrographs for SYTO9/PI staining assay have been deposited to the zenodo (https://zenodo.org/records/14507631).
