# Supplementary material for: Continuous Evolution System Based on Toxin–Antitoxin System Combined with Base Deaminase Accelerates the In Vivo Modification of Target Proteins
Source: Adv Sci (Weinh). 2026 Aug 20;13(59):e76924. doi: 10.1002/advs.76924 (PMC13491524; doi:10.1002/advs.76924)
Supplement: Supplementary file 1 [file ADVS-13-e76924-s001.docx]

**Continuous Evolution System Based on Toxin-Antitoxin System Combined with Base Deaminase Accelerates the *in vivo* Modification of Target Proteins**

Xinyu Zhang^1,2,3,4^, Zhanzhi Liu^1,2,3,4**^[^liu.z@jiangnan.edu.cn^](mailto:liu.z@jiangnan.edu.cn), Ying Xu^1,2,3^, Mingjing Sun^1,2,3^, Guangzhen Zhou^1,2,3^, Deming Rao^1,2,3^, Xiaoqian Chen^1,2,3^, Sheng Wang^1,2,3^, Zhigang Li^1,2,3^, Yan Huang^1,2,3^, Jing Wu^1,2,3*^ [^jingwu@jiangnan.edu.cn^](mailto:jingwu@jiangnan.edu.cn)

1. School of Biotechnology and Key Laboratory of Industrial Biotechnology, Ministry of Education. Jiangnan University, Wuxi, Jiangsu Province, China

2. State Key Laboratory of Food Science and Resources, Wuxi, Jiangsu Province, China

3. International Joint Laboratory on Food Safety, Jiangnan University, Wuxi, Jiangsu Province, China

4. The authors have contributed equally.

Zhanzhi Liu, [liu.z@jiangnan.edu.cn](mailto:liu.z@jiangnan.edu.cn) (ZZL)

Jing Wu, [jingwu@jiangnan.edu.cn](mailto:jingwu@jiangnan.edu.cn) (JW)

**Table S1 The strains used in this study.**

| Strain | Function | Source |
| --- | --- | --- |
| *E. coli* JM109 | Cloning host | Takara Biotechnology Co., Ltd. |
| *E. coli* BL21 (DE3) | Expression and continuous evolution host | Sangon Biotech (Shanghai) Co., Ltd. |
| *E. coli* BL21(DE3)-Test | The native *lacZ* of *E. coli* BL21(DE3) is inactivated by inserting *CmR* | In-house laboratory stock |

**Table S2 The plasmids used in this study.**

| Plasmid | Relevant features | Function |
| --- | --- | --- |
| pET20b | Amp^R^, ColE1 ori, P_T7_ | Expression vector |
| pET24a | Kan^R^, ColE1 ori, P_T7_ | Expression vector |
| pCDFDuet | Sm^R^, CloDF13 ori, P_T7_, double multi-cloning sites | Expression vector |
| pRSFDuet | Kan^R^, RSF ori, P_T7_, double multi-cloning sites | Expression vector |
| MP-AID | Sm^R^, pCDFDuet-P_T7_-*aid*-T7 *rnap* (T3) | Mutagenesis plasmid |
| MP-CT | Sm^R^, pCDFDuet-P_T7_-*Pmcda1-linker*-T7 *rnap* (T3)-P_tac_-*ugi*-P_T7_-*tadA9e-linker*-T7 *rnap* (T3) | Mutagenesis plasmid |
| TP-T7P | Amp^R^, pET20b-P_T3_-T7 *rnap* | Target plasmid containing T7 *rnap* transcribed by T3 promoter |
| TP-T7-GFP | Kan^R^, pET24a-P_T7_-*egfp* | Target plasmid containing *egfp* transcribed by T7 promoter |
| TP-T3-GFP | Kan^R^, pET24a-P_T3_-*egfp* | Target plasmid containing *egfp* transcribed by T3 promoter |
| TP-PsiR | Amp^R^, pET20b-P_T3_-*psir* | Target plasmid containing *psir* transcribed by T3 promoter |
| LP-CC | Kan^R^, pRSFDuet-P_T7_-*ccdA*-araC-P_BAD_-*ccdB* | Lethal plasmid containing gene encoding CcdA/CcdB, T7 promoter/BAD promoter |
| LP-PP | Kan^R^, pRSFDuet-P_T7_-*pezA*-araC-P_BAD_-*pezT* | Lethal plasmid containing gene encoding PezA/PezT, T7 promotor/BAD promoter |
| LP-SS | Kan^R^, pRSFDuet-P_T7_-*sezA*-araC-P_BAD_-*sezT* | Lethal plasmid containing gene encoding SezA/SezT, T7 promoter/BAD promoter |
| LP-EZ | Kan^R^, pRSFDuet-P_T7_-*epsilon*-araC-P_BAD_-*zeta toxin* | Lethal plasmid containing gene encoding epsilon/zeta toxin, T7 promoter/BAD promoter |
| LP-RPP | Kan^R^, pRSFDuet-P_CTGA_-*pezA*-araC-P_BAD_-*pezT* | Lethal plasmid containing gene encoding PezA/PezT as TAS, CTGA promotor/BAD promoter |
| LP-PPG | Kan^R^, pRSFDuet-P_T7_-*pezA-egfp* -araC-P_BAD_-*pezT* | Lethal plasmid containing gene encoding PezA-eGFP/PezT as TAS, T7 promotor/BAD promoter |
| LP-RPG | Kan^R^, pRSFDuet-P_CTGA_-*pezA-egfp*-araC-P_BAD_-*pezT* | Lethal plasmid containing gene encoding PezA-eGFP/PezT as TAS, CTGA promotor/BAD promoter |
| LP-AΔPG | Kan^R^, pRSFDuet-P*_psiAΔ_*-*pezA-egfp*-araC-P_BAD_-*pezT* | Lethal plasmid containing gene encoding PezA-eGFP/PezT as TAS, psiAΔ promotor/BAD promoter |

**Table S2 (Continued)**

| Plasmid | Relevant features | Function |
| --- | --- | --- |
| pCDFDuet-P_CTGA_-*gal* | Sm^R^, identification vector containing the gene encoding *Bacillus circulans* ATCC 31382 β-galactosidase | Identification and characterization of T7 RNAP variants, CTGA promoter |
| pSB1C3-P*_tac_*-*psir*-P*_psiAΔ_*-*memerald* | Cm^R^, identification vector containing the gene encoding Green fluorescent protein mEmerald | Identification and characterization of PsiR variants, psiAΔpromoter |

**Table S3 The primers used in this study.**

| Name | Sequence (5'-3') | Function |
| --- | --- | --- |
| CTL OS-F | GTTATAACTCGAGTCTGGTCGGCCGCATAATCGAAATTAATAC | MP-CT construction |
| CTL OS-R | CGCAGCAGCGGTTTCTTTGCGGCCGCAAGCTTTTAC | MP-CT construction |
| CL OS-F | GCAAAGAAACCGCTGCTGC | MP-CT construction |
| CL OS-R | CCGACCAGACTCGAGTTATAAC | MP-CT construction |
| P_T3_ SDM-F | CCCGCGAAATTAATAACCCTCACTATAGGGAGA | Replace PT7 with PT3 |
| P_T3_ SDM-R | TCTCCCTATAGTGAGGGTTATTAATTTCGCGGG | Replace PT7 with PT3 |
| T7P-F | CTTTAAGAAGGAGATATACATATGAACACGATTAACATCGC | TP-T7 RNAP construction |
| T7P-R | TGGTGGTGGTGGTGCTCGAGTTACGCGAACGCGAAGTCC | TP-T7 RNAP construction |
| PsiR-F | CTTTAAGAAGGAGATATACATATGACCGGTATCTCTTCTAAAAAAGC | TP-PsiR construction |
| PsiR-R | TCTCAGTGGTGGTGGTGGTGGTGCTCGAGTTTAGCCATACGACGCAGAGCAAC | TP-PsiR construction |
| T7PJ-F | CTCACTATAGGGAGACCACAACGG | TP-T7 RNAP identification |
| T7PJ-R | GTTAGCCCAATAGCCACCACC | TP-T7 RNAP identification |
| araBAD-F | GAAAGTAATCGTATTGTACACGTGTCAAATGGACGAAGCAGGGAT | LP construction |
| araBAD-R | TGCAATATGGACAATTGGTTTCTTCTCTGAATGGCGGGAGTATGAAAAG | LP construction |
| PCTGASDM-F | TTAGGAAATTAATACCTGACACTATAGGGGAATT | Replace PT7 with PCTGA |
| PCTGASDM-R | AATTCCCCTATAGTGTCAGGTATTAATTTCCTAA | Replace PT7 with PCTGA |
| LG-F | ACGTATGGTCGCAGGTGGTGGTGGTAGCGGTGGTGGTGGTAGCATGTCCCGTGTATCA | LP-PPG/LP-RPG construction |
| LG-R | GCATTATGCGGCCGCAAGCTTTTACTTGTACAGTTCATCCATACCCA | LP-PPG/LP-RPG construction |
| PA-F | GCGACTCCTGCATTAGGAAATGTATAAATGGTGGCTTTTTTTGAACTTATGCCCGTC | LP-AΔPG construction |
| PA-R | CATTTGTACCCATGGCTGCTGCAACCACCATGCACAATC | LP-AΔPG construction |
| Bc-F | ATCATCATCATCATCATGGTATGGGTAACTCTGTTTCTTACGATGGTG | pCDFDuet-P_CTGA_-*gal* construction |
| Bc-R | TCTCATCCGCCAAAACAGCCAAGCTTGTCGACTTACGGGGTAAC | pCDFDuet-PCTGA-*gal* construction |
| GNGS-F | ATGTCCCGTGTATCAAAAGGG | PCR for NGS |
| GNGS-R | GTGACCCAAGATATTCCCGTC | PCR for NGS |

**Table S3 (Continued)**

| Name | Sequence (5'-3') | Function |
| --- | --- | --- |
| GFP-F | GATCTTCCCCATCGGTGATGT | Sanger sequencing |
| T7PS-F | GTCCTCAACGACAGGAGCACGATCA | Sanger sequencing |
| T7PS-R | GCAGCCAACTCAGCTTCCTTTC | Sanger sequencing |

**Table S4 The substitutions of the promising T7 RNAP variants.**

| system | Amino acid change | Original codon | Existing codon | Mutation |
| --- | --- | --- | --- | --- |
| PEACE1.0 | R627G | CGC | GGC | C to G |
|  | R627A | CGC | GCC | C to G, G to C |
|  | R627V | CGC | GTC | C to G, G to T |
|  | R478H | CGC | CAC | G to A |
| PEACE2.0 | T76S | ACC | GCC | A to G |
|  | A115V | GCG | GTG | C to T |
|  | A413T | GCC | ACC | G to A |
|  | Y458C | TAC | TGC | A to G |
|  | A519V | GCT | GTT | C to T |
|  | P451L | CCA | CTA | C to T |
|  | R478C | CGC | TGC | C to T |

**Table S5 The substitutions of the promising PsiR variants.**

|  | Amino acid change | Original codon | Existing codon | Mutation |
| --- | --- | --- | --- | --- |
| PsiR-M | G285D | GGT | GAT | G to A |
| PsiR-2M | G285D/C286Y | GGT/TGC | GAT/TAC | G to A, G to A |
| PsiR-3M1 | G285D/C286Y/F315F | GGT/TGC/TTC | GGT/TGC/TTT | G to A, G to A, C to T |
| PsiR-3M2 | G285D/C286Y/E316K | GGT/TGC/GAA | GGT/TGC/AAA | G to A, G to A, G to A |
| PsiR-3M3 | G285D/C286Y/E309E | GGT/TGC/CAG | GGT/TGC/CAA | G to A, G to A, G to A |
| PsiR-4M | G285D/C286Y/F292L/R322R | GGT/TGC/  TTC/CGT | GGT/TGC /  CTC/CGC | G to A, G to A, T to C, T to C |

**Figure S1. Mutation efficiency characterization of MP-AID.** After continuous culture of *E. coli* BL21(DE3)/MP-AID/TP-T3-GFP and *E. coli* BL21(DE3)/TP-T3-GFP, NGS with approximate 2.4×10^4^ reads is used to analyze the gene fragments of the eGFP variants sorted through FACS, and the mutation efficiency of each site is calculated and illustrated, which is shown in red and blue, respectively.

**Figure S2. Transcriptional activity of T7 RNAP wild type toward P_CTGA_.** “▼” means no inducer, “●” means IPTG only, inducing antitoxin expression (I/-), “▲” means *l*-Arabinose only, inducing toxin expression (-/A), and “■” means Both IPTG and *l*-arabinose, co-inducing antitoxin and toxin expression (I/A).

**Figure S3. The impact of high-level expression of MP-AID on host growth.** “PEACE” means the inducer is IPTG, “CX1” means no inducer, “CX2” means the inducer is IPTG, and no MP-AID. Every 24 hours, the bacterial culture is transferred to fresh medium and further cultivated under the same conditions.

**Figure S4. Selection results for T7 RNAP evolution.** “PEACE”: the experimental group that underwent normal evolution with MP‑AID induction, “CX1” means without MP‑AID induction, “CX2” means lacking the mutagenesis plasmid. Equal volumes of cultures were spread onto plates containing the toxin inducer: the PEACE group was plated with a cell density of 0.01 (OD_600_), CX1 with 0.1 (OD_600_), and CX2 with 0.01 (OD_600_).

**Figure S5. Mutation efficiency characterization of MP-CT.** After continuous culture of *E. coli* BL21(DE3)/MP-CT/TP-T3-GFP and *E. coli* BL21(DE3)/MP-T3P/TP-T3-GFP, Kbseq was used to analyze the eGFP variant gene fragments sorted out by FACS, and the mutation efficiency of each site was calculated and explained, respectively represented in red and blue.

**Figure S6. FACS screening results for T7 RNAP evolution using PEACE 2.0.** Due to the large number of experimental groups, the data are split into two side-by-side panels for visual clarity, with each panel labeled “T7 RNAP variants sorting” at the top left. Histograms show the distribution of eGFP fluorescence intensity across multiple independent evolution runs. The x axis indicates eGFP fluorescence intensity (a.u.), and the y axis indicates cell count. To emphasize the positive population, only the region above 10^4^ a.u. threshold is displayed. Overlaid semi-transparent histograms from individual runs illustrate the consistency of screening outcomes across replicates.

**Figure S7. Transcriptional activity of PsiR wild type toward P*_psiAΔ_*.** “▼” means no inducer (-/-), “●” indicates the inducer is *d*-allulose (Al/-), inducing antitoxin expression, “▲” means *l*-arabinose as inducer, inducing toxin expression (-/A), and “■” indicates that *d*-allulose and *l*-arabinose are added as inducers, co-inducing antitoxin and toxin expression (Al/A).

**Figure S8. Screening for PsiR mutants. a** FACS analysis results of PsiR mutants. “WT” indicates the unmutated control group; “Mut.1-3” indicates the parallel group set during continuous evolution. **b** Analysis of the response of PsiR mutants to different sugars in microtiter plates. The abscissa is the strain number, and the red box is the control group that has not undergone continuous evolution.
